# Theta and alpha connectivity in children with autism spectrum disorder

**DOI:** 10.1093/braincomms/fcaf084

**Published:** 2025-02-19

**Authors:** Samuel J K Barnes, Megan Thomas, Peter V E McClintock, Aneta Stefanovska

**Affiliations:** Department of Physics, Lancaster University, Lancaster LA1 4YB, UK; Department of Paediatrics, Blackpool Teaching Hospitals NHS Foundation Trust, Blackpool FY3 8NR, UK; Department of Pediatrics, Faculty of Medicine, Dalhousie University, Halifax, Nova Scotia, Canada NS B3H 4R2; Department of Physics, Lancaster University, Lancaster LA1 4YB, UK; Department of Physics, Lancaster University, Lancaster LA1 4YB, UK

**Keywords:** electroencephalogram, neural connectivity, phase coherence, wavelet, coupling

## Abstract

Spontaneous electroencephalography (EEG) measurements have demonstrated putative variations in the neural connectivity of subjects with autism spectrum disorder, as compared to neurotypical individuals. However, the exact nature of these connectivity differences has remained unknown, a question that we now address. Resting-state, eyes-open EEG data were recorded over 20 min from a cohort of 13 males aged 3–5 years with autism spectrum disorder, and nine neurotypical individuals as a control group. We use time-localized, phase-based methods of data analysis, including wavelet phase coherence and dynamical Bayesian inference. Several 3 min signal segments were analysed to evaluate the reproducibility of the proposed measures. In the autism spectrum disorder cohort, we demonstrate a significant (*P <* 0.05) reduction in functional connectivity strength across all frontal probe pairs. In addition, the percentage of time during which frontal regions were coupled was significantly reduced in the autism spectrum disorder group compared to the control group. These changes remained consistent across repeated measurements. To further validate the findings, an additional resting-state EEG dataset (eyes open and closed) from 67 individuals with autism spectrum disorder and 66 control group individuals (male, 5–15 years) was assessed. The functional connectivity results demonstrated a reduction in theta and alpha connectivity on a local, but not global, level. No association was found with age. The connectivity differences observed suggest the potential of theta and alpha connectivity as biomarkers for autism spectrum disorder. Additionally, the robustness to amplitude perturbations of the methods proposed here makes them particularly suitable for the clinical assessment of autism spectrum disorder and of the efficacy of therapeutic interventions.

## Teaser

Connectivity within the theta and alpha bands is reduced in boys with autism, according to Barnes *et al*. Their phase-based functional and effective connectivity analyses of EEG data are robust against amplitude perturbations through movement artefacts and noise. It promises reliable clinical evaluation of children with autism, even during movement.

## Graphical Abstract

**Graphical Abstract:**
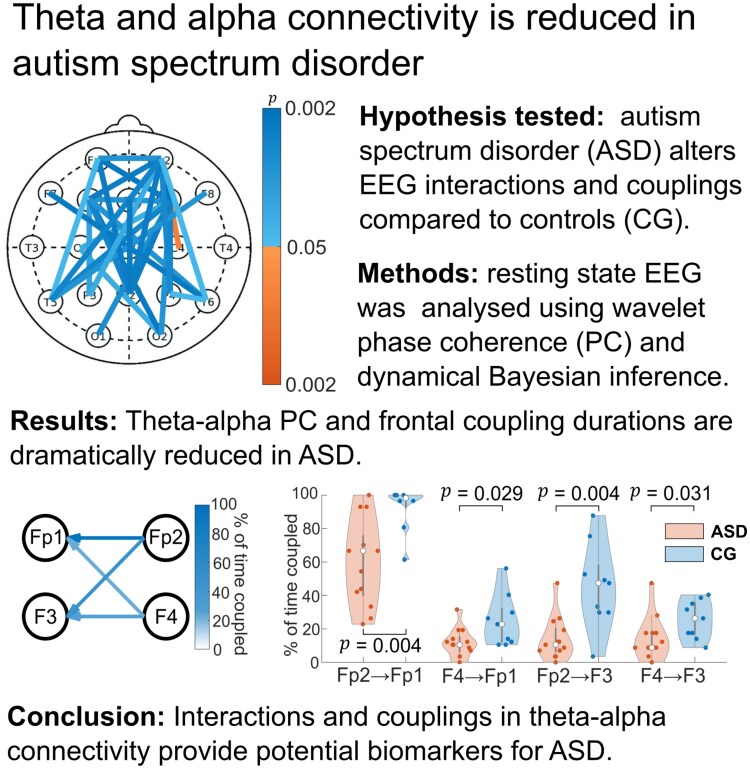

## Introduction

Altered neural connectivity is a hallmark of autism spectrum disorder (ASD).^1-7^ In general, a prevailing pattern of hypoconnectivity in the low- and medium-frequency bands, alongside over-connectivity in the high-frequency intervals, has been observed.^8^ Concurrently, the role of temporal regulation in maintaining couplings between brain regions has become apparent in a wide range of neurological conditions including ASD.^9-11^ Despite this, previous results exhibit considerable variability.^12^ Particular inconsistencies pertain to the balance between long- and short-range connections in ASD,^8,13-16^ and the relative hyperconnectivity^17,18^ or hypoconnectivity^16,19-21^ of frontal regions. These discrepancies can be attributed partially to the heterogeneity of ASD populations,^22^ variations in measurement approaches^23,24^ and alternative data analysis methodologies.^25^ Additionally, the duration of observation may have been insufficient to reveal couplings. Detecting coupled oscillatory behaviour requires the observation of at least thirty cycles, and is therefore dependent on the frequency of interest. This requirement is particularly relevant for EEG data, which is often highly nonlinear, non-autonomous, and non-stationary, leading to fluctuations in frequency content over time.^26^

When measuring brain activity in children, movement artefacts are inevitable and should be anticipated and accounted for. Artefact-resilient approaches are therefore necessary for findings to be reliable and appropriate for clinical application. The methods employed in this study focus on phase, rather than amplitude, dynamics. By also focusing on finite-time dynamics, our approach acknowledges the non-autonomous nature of underlying oscillatory modes and reduces the effects of amplitude perturbations on measured connectivity. Additionally, our method detects nonlinear interactions, overlooked by linear, statistical approaches.^27,28^ The wavelet transform enables an optimal trade-off between the time-localization and frequency resolution, avoiding the bias in Fourier based approaches.^29^ Furthermore, our approach^26,30^ enables the simultaneous detection of phase differences across multiple modes within a frequency band, in contrast to methods that reduce the phase to a single value.^31-33^ Taken together, these features enable the present approach to provide an interpretable way of uncovering deterministic oscillations within multiscale, nonlinear and time-varying data.

In recent years, there has been a growing emphasis on adopting effective connectivity methods for assessing brain network dynamics.^1,34^ Effective connectivity approaches can capture the directionality of couplings within the brain whilst also enabling the inference of causal relationships between different regions. In contrast, functional connectivity methods may mistake a common external factor for mutual interaction.^35^ Novel approaches that incorporate directionality and infer causality may offer a more comprehensive view of the complex interactions occurring within the brain. Living systems are inherently non-autonomous. Therefore, when evaluating time series of biological origin, time should be treated as an explicit physical parameter.^36^ To account for underlying temporal variability, here effective connectivity is calculated over a sequence of data windows. The duration of couplings may then be calculated to assess their presence over a larger time interval. The non-autonomous nature of neural couplings, which time-asymptotic approaches would disregard,^30,37^ may thereby be captured.

Individuals with ASD often exhibit deficits in executive function (EF), manifesting as several core challenges.^21,38-40^ Previous research has associated frontal areas of the brain with EF,^41-43^ and ASD individuals exhibit structural differences in these regions, such as minicolumnar abnormalities^44^ and brain overgrowth.^45,46^ Additionally, an imbalance of GABA and glutamate has been identified, prompting pharmacological interventions that have enhanced prefrontal connectivity in ASD individuals.^47^ Multiple studies have also reported decreased inter-hemispheric connectivity in ASD^48-51^ particularly in homotopic regions.^52^

We hypothesize that, using our novel approach to data analysis, we can quantify the deficits in frontal inter-hemispheric connectivity and characterize their time-variations. Our results demonstrate a significant reduction in alpha and theta functional connectivity in children with ASD. It is hoped that this approach will provide a valuable tool in the quest for quantitative biomarkers characterizing ASD.

## Materials and methods

Fourteen males aged 3–5 years with diagnoses of ASD were identified through Blackpool Teaching Hospitals NHS Foundation Trust’s Child Development Centre. Ethical approval was obtained from the NRES Committee North West — Lancaster REC, reference number: 13/NW/0509. Participants’ parents provided informed consent, and the clinical study was registered as UKCRN ID14936. Ten age-matched neurotypical controls were recruited through advertisements at Health and University sites and nurseries. One individual in each group was unable to complete the full recording. Inclusion criteria were either a clear diagnosis of an ASD, confirmed by an Autism Diagnostic Observation Schedule (ADOS) assessment; or no concerns about development or features of ASD, confirmed by a developmental history and ADOS assessment. Exclusion criteria were epilepsy or undiagnosed seizure episodes; medications known to affect brain function; structural brain abnormalities; chromosome abnormalities; and, for the control group only, a first-degree relative with ASD diagnosis. Initially, the study aimed to recruit a similar number of female participants. However, due to the lower incidence of diagnosed ASD in females, it was not possible to recruit enough girls with an ASD diagnosis within the study’s time frame. A small age range was selected to control for developmental changes. Based upon the sizes of the ASD and CG groups, sensitivity analysis was performed using G*Power. Due to the sample sizes used in this study, effect sizes of 1.387 were able to be reliably detected. A full summary, including further details of the sensitivity analysis, is given in the Supplementary material. The age, ADOS score and hand preference of each participant are provided in Table 1.

**Table 1:** Participant details

Subjects	Age (months)	ADOS score	Hand	Setting		Activity			State		
ASD	Mean: 50 ± 6	Mean: 17.6 ± 3.5	R: 53.8% L: 7.7% ND: 38.5%	Lap	Chr	Rest	Screen	BBles	Slp	Qt	SM
1	42	19	ND	X		X			X		
2	45	19	ND	X				X			X
3	50	19	R	X			X			X	
4	56	11	ND	X		X				X	
5	50	22	ND	X		X				X	
6	50	20	ND	X		X				X	
7	52	20	R		X		X			X	
8	47	21	ND		X	X				X	
9	56	13	L		X	X				X	
10	43	19	ND	X			X			X	
11	58	16	R	X			X			X	
12	56	18	R	X			X			X	
13	58	12	R	X		X				X	

CG	Mean: 46 ± 7	Mean: 1 ± 1	R: 55.6%L: 0%ND: 44.4%								
1	55	0	R	X			X		X		
2	45	2	R		X	X				X	
3	53	0	R		X		X			X	
4	55	0	ND	X		X				X	
5	44	2	ND	X		X					X
6	44	0	R	X		X				X	
7	47	1	R	X		X				X	
8	36	2	ND	X		X					X
9	36	2	ND	X		X				X	

The state of each participant during the measurements for each group. The first four columns give summary data: ASD (autism spectrum disorder) or CG (control group) subject number; age in months; ADOS (autism diagnostic observation schedule) score; and right (R) or left (L) or not-defined (ND) handedness. The next two columns show whether the participant was sitting on their parents’ lap (Lap) or on a chair (Chr). The next three columns show what the participant was doing during the recording, either at rest (Rest), or watching a screen (Screen), or watching bubbles (Bbles). The final three columns indicate their level of activity during the recording, whether sleepy (Slp), quiet (Qt), or exhibiting some small movements (SM). All participants were male. Details regarding effect size are given in the Supplementary material.

### Data recording

EEG signals were recorded using a Nicolet cEEG instrument (Viasys Healthcare, USA) at a sampling rate of 256 Hz, with 19 probes and one reference electrode placed on the child’s scalps using the standard 10–20 configuration. EEG was recorded for 20 min while the child was in an eyes-open resting condition and sitting on a chair, their stroller, or their parent’s lap. When necessary to maintain a relaxed state, participants were presented with soap bubbles or smartphone videos. Details for individual participants are provided in Table 1. A 3 min segment was selected from the data, based on a video of the child recorded during the data collection. It was chosen as being the interval where the child appeared to move the least. Henceforth, this shall be referred to as the ‘video’ segment. As this segment was expected to have minimal movement artefacts, it provided a reliable starting point for the initial investigation. A retest procedure was employed to assess the robustness of the findings and account for the real-world conditions encountered in clinical settings. Five additional segments, each lasting 3 min, were chosen by visual inspection of the data, aiming to avoid segments with the largest spikes in the time series. Details regarding the pre-processing of the data are given in the Supplementary material.

### Additional dataset

An additional dataset was acquired to further validate the results. The Healthy Brain Network (HBN) biobank contains multimodal brain imaging datasets complemented by a wide range of phenotypic data.^53^ The recruitment procedure utilized a community-referred model, in which advertisements invited concerned parents and caregivers to seek diagnosis and support for their children if needed. From this neurodiverse cohort, several children were diagnosed with ASD, while a group of individuals were given no diagnosis and acted as a control group. Age, gender, IQ and handedness were matched between the groups compared. Statistical descriptions of each group’s composition are given in Table 2.

**Table 2:** Median values and significances between groups in the HBN data

Groups	ASD (*n* = 67)			CG (*n* = 66)			Group comparison
	Median	SD	Range	Median	SD	Range	
Age (years)	8.73	2.58	9.61	8.99	2.76	9.95	0.721
IQ	100	17.3	84.0	104	11.8	68.0	0.0517
Handedness	68.4	58.3	198	86.7	51.8	196	0.0529
SRS	85	30.9	114	26	15.8	81	4.07 × 10^−19^

The median, standard deviation (SD) and range of age, IQ, handedness and social responsiveness score (SRS) in the ASD and CG groups. All participants were male. The Wilcoxon rank-sum test was used to evaluate the differences in potentially confounding factors between groups; none of which were significant apart from the SRS. Handedness was evaluated using the Edinburgh Handedness Questionnaire, with 100 being right hand dominant, 0 ambidextrous and −100 implying left-hand dominance.

The HBN data had been recorded using a 128-channel EEG Geodesic Hydrocel System by Electrical Geodesics Inc. (EGI) sampled at 500 Hz with a bandpass of 0.1–100 Hz. The resting state measurement procedure entailed viewing a fixation cross on a computer screen, with eyes sequentially opened and closed in 20/40 s repeats.^53^ The recordings lasted 5 min, and the central 3 min were selected for analysis. Except where stated otherwise, the same procedures and analysis were applied to both datasets. The results and discussion presented in this paper generally describe the Blackpool data, with the HBN dataset being treated as a validation dataset.

#### Frequency bands

For three reasons, we chose to merge the theta–alpha range into a single frequency interval (3.5–12 Hz). First, the limits of the five traditional frequency bands (delta, theta, alpha, beta and gamma) were not clearly distinguishable in the power spectra of the EEG data. Thus, it was not possible to draw clear boundaries between the alpha and theta bands for any given subject without crossing peaks in the spectra of other subjects. Secondly, it has been found that EEG power spectra evolve during developmental years as children’s brains undergo maturation of cellular substrates.^54,55^ Specifically, alpha activity has been found to occur at lower frequencies (∼8 Hz instead of ∼10 Hz) in younger children.^54,56^ Finally, previous studies of EEG activity in children with ASD do not use strict limits for their frequency bands, and no standard has been established to date.

### Data analysis

Rate processes are ubiquitous in nature. From celestial motion to cellular metabolism, oscillatory behaviour is pervasive across all spatial and temporal scales of existence. The brain is no different, with electrical activity that propagates information between regions, often taking certain characteristic frequencies dependent on the information it conveys.^57^ The exceedingly high connectivity between neurons gives rise to networks of oscillators. EEG signals are generated by transmembrane ion currents in the pyramidal neurons of the cortex and are transmitted to electrodes on the scalp via volume conduction.^58^ The nature of these signals is inherently oscillatory. To elucidate their underlying behaviour, we focus on time-resolved oscillatory modes present in the recorded EEG signals.

#### Time-frequency representation

The first step in identifying these modes is to explore their presence in the time-frequency domain. The wavelet transform can reveal potential non-autonomicity within oscillations.^59^ Additionally, the logarithmic frequency resolution offered by this method provides a more balanced distribution of information across frequency bands. The time-frequency representation is generated by sliding wavelets of varying scales along the input signal and transforming the overlapping parts into the frequency domain. Lower frequencies exhibit fewer oscillations within a given time, necessitating a larger scale to capture the oscillatory activity effectively. Conversely, higher frequencies require less time to be captured, allowing the use of a smaller scale. This optimizes the trade-off between time-localization and frequency resolution, providing greater time-localization at high frequencies and improved frequency resolution at low frequencies. The wavelet transform was therefore chosen for the initial analysis of these signals. It is defined by


(1) $$W_{T}(s,t)=\int_{-L/2}^{L/2}\;\Psi (s,u-t)f(u)du,$$

where the mother wavelet, Ψ(s,t), is the object contracted and dilated to reveal oscillatory behaviour at various times and scales. For the present study, the Morlet mother wavelet was used,


(2) $$\Psi (s,t)=\frac{1}{\sqrt[{4}]{\pi }}\left(e^{\frac{2\pi i\omega_{c}t}{s}}-e^{-\frac{2{\pi \omega }_{c}^{2}}{2}}\right)e^{-\frac{t^{2}}{2s^{2}}},$$

which is composed of a sinusoidal wave within a Gaussian envelope.^60^ The frequency of the sinusoid allows one to focus upon a given scale, while the Gaussianity ensures a smoothly decaying amplitude and, therefore, greater time-localization. The enhanced temporal resolution, and its common use in neuroscience,^61^ motivated the selection of this wavelet.

In contrast, linear approaches, such as those based on the Fourier transform, often use a fixed window size when evaluating the time-frequency domain. This fixed window length leads to suboptimal multiscale analysis. Furthermore, linear methods often reduce the amount of information captured by averaging over time, or filtering out potentially deterministic oscillations that are falsely categorized as noise.^30^ A cornerstone of the present approach is the maximal preservation of the underlying information.

**Figure 1:**
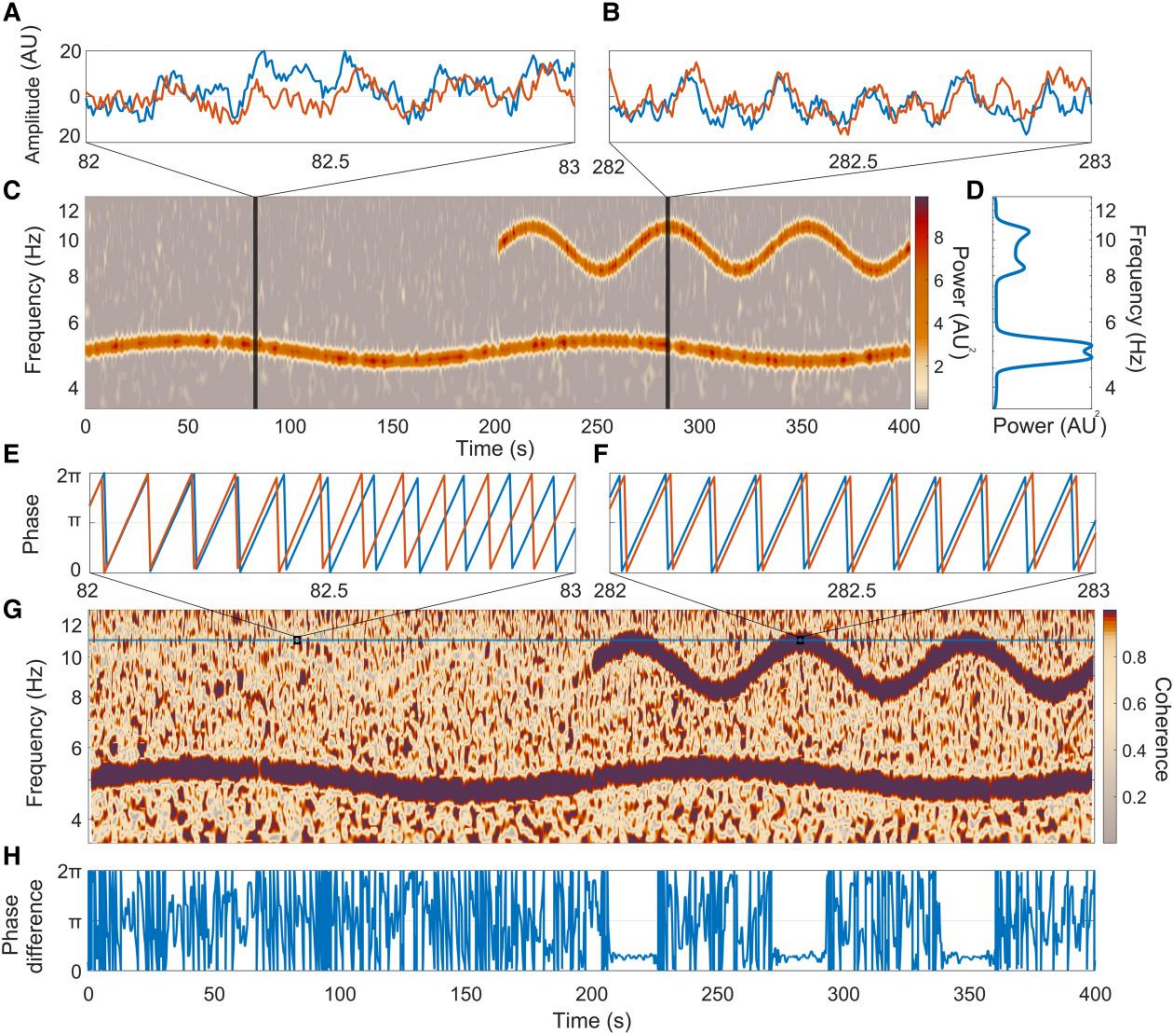
**Demonstration of wavelet phase coherence using a pair of simulated time series.** (**A**). A 1s-long window taken from the first half of the simulated time series. Further details of the modelling procedure and parameters are given in the Supplementary material. (**B**) A 1s-long window taken from the second half of the simulated time series. (**C**) Time-frequency representation of the warmer coloured (orange) time series’ power calculated using the wavelet transform (Morlet wavelet, frequency resolution = 4). (**D**) Time-averaged power of the warm coloured (orange) series. The frequency axes of (**C**) and (**D**) are logarithmic. (**E–H**) A step-by-step evaluation of wavelet phase coherence. (**E**, **F**) The phases of the time series, calculated at 11 Hz, for intervals corresponding to those in **A** and **B**. (**G**) Time-localized phase coherence between the warm (orange) and cooler coloured (blue) time series. The horizontal line at 11 Hz indicates where the phases (**E**, **F**) and phase difference (**H**) were evaluated. (**H**) The phase difference at 11 Hz is almost flat across times that correspond to high time-localized coherence values.

The wavelet transform generates a complex matrix containing both phase and amplitude information, facilitating a comprehensive multiscale analysis across time. This dual representation captures the magnitude of specific frequencies while also elucidating the temporal evolution of oscillatory modes, enabling a more nuanced understanding of the underlying dynamics. A simulated example signal (Fig. 1A and B) containing two oscillatory modes at different frequencies is analysed in Fig. 1C and D. It is evident that the wavelet approach readily elucidates that there are two modes with time-varying frequencies representative of alpha and theta oscillations. The time-localization and logarithmic frequency resolution reveal the non-autonomous nature of the oscillatory modes.

When considering non-autonomous dynamics, time-averaged approaches may not always faithfully represent the number of oscillatory modes present in the signal. For example, Fig. 1D in isolation may naively give the impression that additional oscillatory modes are present. The time-frequency domain, as illustrated in Fig. 1C, reveals, however, that this is not the case. The false identification of modes is a common mischaracterization^30^ resulting from a failure to consider the time domain. Once the oscillatory modes have been identified, functional connectivity methods may then be applied to ascertain dependences between the respective oscillators.

#### Wavelet phase coherence

The wavelet transform assigns phase values to each point in the time-frequency domain for the underlying oscillatory modes. Following this, wavelet phase coherence (WPC) is calculated to determine the interaction between a pair of signals and how it develops over time.^62-65^ The consistency of the phase difference between the signals at each point in the time-frequency domain is evaluated across several (in the present case 10) complete oscillations at a given frequency, *f*,


(3) $$\mathrm{WPC}t,f=\frac{\;f}{10}\left|\int_{t-\frac{5}{f}}^{t+\frac{5}{f}}\;e^{i(\phi_{s,f}^{1}\;-\;\phi_{s,f}^{2})}ds\right|.$$

Subsequently, a value is allocated between zero (complete lack of coherence) and unity (perfect coherence—where the difference in the phases $\left(\phi_{s,f}^{1}-\phi_{s,f}^{2}\right)$ at each point in the time-frequency domain remains constant over the time interval). The WPC is completely independent of amplitude dynamics and depends purely on the phase of the oscillations. By only considering phase dynamics, this approach is more resilient to movement artefacts and noise than its amplitude-weighted counterpart.^26^

An illustrative example demonstrating time-localized WPC is depicted in Fig. 1A–H. Two model time series are represented by the blue and orange lines respectively in Fig. 1A and B. Specific details of the modelling procedure used to generate these time series are outlined in the Supplementary material. To summarize, the orange line lacks the high-frequency (HF) mode for the first 200 s (see Fig. 1C for the time-frequency representation of the orange line), the blue line is modelled similarly, but with the HF mode lasting the entire 400 s. Independent realizations of white Gaussian noise were applied to both of the simulated signals. Consequently, there is no shared high frequency oscillatory component between these signals for the first 200 s, resulting in a continuously changing phase difference (Fig. 1E). This variability in phase difference is further depicted in the time-localized phase coherence in Fig. 1G.

To contrast this inconsistency between the phases of the HF mode in the first half of the signal, coherent signals were generated in the subsequent half; establishing shared behaviour between the time series during this interval. WPC detects this shared oscillatory component by examining the phase difference between the oscillations. The phase difference at 11 Hz (Fig. 1H) is constant during intervals of high time-localized coherence (Fig. 1G).

#### Global coherence

The wavelet mean field was used to evaluate global coherence across the brain. For *N* time series evaluated simultaneously at different probe locations, we have,


(4) $$r_{\sigma }(t)=(1/N)\sum_{n=1}^{N}w_{n,\sigma }(t),$$

where,


$$w_{n,\sigma }(t)=W_{n,\sigma }(t)/\sqrt{(1/NT)\sum_{n=1}^{N}\sum_{t=1}^{T}W_{n,\sigma }(t)\bar{W_{n,\sigma }(t)}}.$$

The corresponding wavelet transforms across time and scale are represented as $W_{n,\sigma }(t)$, with the overbar denoting complex conjugation. When the time series exhibit similar phases at a given time and frequency, the average $\sum_{n=1}^{N}w_{n,\sigma }(t)$ will yield a large complex number due to the reinforcement of synchronized oscillations. Conversely, when the probe signals are unsynchronized, the phasors will point in random directions in the complex plane and cancel. The degree of interaction across the brain is calculated with the mean-squared magnitude of the wavelet mean field, $(1/T)\sum_{t=1}^{T}{\left|r_{\sigma }(t)\right|}^{2}$. This takes a value between zero and unity, where 1 represents complete synchrony across the brain and 0 is perfect desynchronization.^66^

#### Dynamical Bayesian inference

**Figure 2:**
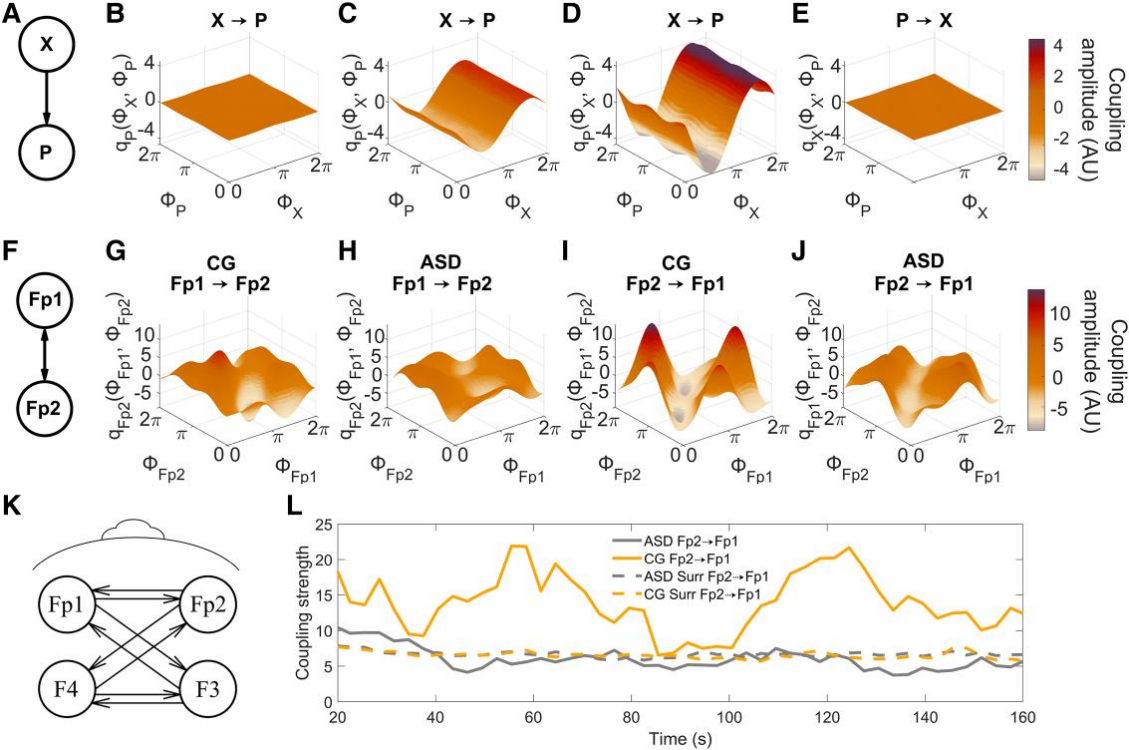
**Dynamical Bayesian inference applied to a model and measured data.** (**A**) A model of the unidirectional coupling X → P, where the coupling functions (q) depend upon the phases (ϕ) of each oscillator. Further details of the modelling procedure and parameters are given in the Supplementary material. (**B–D**) Coupling functions at different strengths (E = 0, 5 and 10, respectively. (**E**) Coupling in the opposite direction (P → X) simulated with E = 10, illustrating the unidirectional nature of the coupling. (**F**) Bidirectional neural coupling measured between two probes, Fp1 and Fp2. (**G–J**) Time-averaged coupling functions in an individual with autism spectrum disorder (ASD—**H**, **J**) and a control group individual (CG—**G**, **I**). (**K**) The frontal network couplings evaluated in the present study. (**L**) An example of the coupling strength from Fp2 to Fp1 in a randomly selected individual with ASD (cool colour, grey) and a CG participant (warm colour, gold). Surrogate values are indicated by the dashed lines (95th percentile, intersubject surrogates).

Functional connectivity methods, such as those outlined above, identify statistical dependences between different brain regions.^35^ While this is helpful in highlighting differences between groups, it is essentially a descriptive measure of the common behaviour between a pair of time series. Further, although high values of functional connectivity may reflect underlying neural connections, they may also arise due to a common external influence. Effective connectivity goes beyond this by explicitly describing the influence that one neural region exerts over another. In this way, the strength and direction of influence between regions can be described and quantified. This information comprises the coupling between systems, and the coupling function (CF) describes the way in which information is propagated from one oscillator to another.^67^ Dynamical Bayesian inference^68^ (DBI) was chosen to detect phase couplings between the probes. To appreciate the utility of CFs, consider a pair of unidirectionally coupled phase oscillators,


(6) $$\;\phi_{X}=\omega_{X},\;$$

$$\phi_{P}=\omega_{P}+q_{P}(\phi_{X},\phi_{P})=\omega_{P}+E\;\cos(\phi_{X}+\pi /2.5),$$

where the phase of oscillator *X* (*ϕ_X_*) modulates the phase of *P* (*ϕ_P_*) according to the behaviour of a CF *q_P_*($\phi_{X}$, $\phi_{P}$). As an illustration, this system (Fig. 2A) was simulated numerically, and DBI was applied to reconstruct the coupling functions over a range of coupling strengths. The couplings are represented in Fig. 2B–E, with varying amplitudes, demonstrating that in addition to reconstructing the shape of CFs, DBI can also infer the coupling strength. Directionality may also be captured using this approach. In Fig. 2E, the coupling function is almost zero as there is no information flow from *P* to *X*; thus, via the inference of and comparison between CFs, one can also deduce the direction of an interaction. Real data are also used to demonstrate the bidirectional nature of the couplings under investigation (Fig. 2G–J).

By use of a sliding time window, DBI can evaluate the presence and time evolution of couplings between signals, and so can detect whether the phase behaviour at one location is influencing that at another. DBI is based on Bayes’ theorem and so uses prior knowledge of the parameters of a system to evaluate its current state. Time-evolving dynamics can then be inferred as the information is propagated between windows. Mathematical details of this approach were given earlier.^68-70^ Importantly, as the coupling is assessed over a sequence of windows, its dynamical evolution may be investigated. Even at rest, the influence of brain regions over one another changes with time, so that asymptotic approaches may inadvertently average out interesting transient phenomena. Furthermore, quantifying the coupling strength between probes may lead to false conclusions when averaging over time. A high coupling strength averaged across the entire interval might lead to the incorrect conclusion that the coupling remained consistent throughout. In reality, it could be attributed to one isolated, exceptionally high value in a single window. To avoid this problem, we instead measure the percentage of time during which probes remain coupled. The dynamical nature of this coupling is indicated by Fig. 2L. Here, the dashed lines represent the respective intersubject surrogates, while the solid lines represent the coupling strengths of randomly chosen CG and ASD individuals. In this way, the amount of time spent in a coupled/decoupled state is calculated. In the present study, a window size of 3 s was selected, as it contained at least 10 cycles of the slowest oscillation of interest (10/3.5 = 2.8). The propagation constant was set at 0.2 to control the information transferred between windows, and the overlap parameter was set to unity (no overlap).

### Statistical analysis

#### Testing for significance using surrogate data

Surrogate analysis was used to assess the significance of results. The underlying goal is to generate a surrogate time series with similar statistical properties to the original time series, but with randomized phase evolution. Any coherence found to be significantly lower than that produced between uncorrelated time series is treated as non-significant. A significance threshold is set, based on the surrogate values. Different types of surrogate data have been discussed.^71^

For any pair of probes, the time-averaged (mean) coherence was calculated between the respective time series of all subjects in each group. For example, in the ASD group, for probe pair F3–F4, coherence was calculated between the F3 time series of 13 subjects and the F4 time series of the same 13 subjects. This results in a set of 13 × 13 overall coherence values. The 13 diagonal values were the actual coherence values for a given person between the probe pair F3–F4, while the remaining 156 were the ‘apparent’ coherence between different subjects’ signals (whereas, in reality, there could not have been any coherence). First, the mean of the 156 surrogate coherence values was calculated for each frequency within the frequency band of interest. This was treated as a threshold and subtracted from the actual coherence to give the net, or effective, coherence. Any values below the surrogate threshold following this subtraction were set to zero. The mean coherence was then calculated as a single value for each participant and each probe combination. For the DBI analysis, the 95th percentile served as a surrogate threshold because the results were systematically higher for coupling time.

#### Statistical tests

Following the application of surrogate testing, statistical tests were applied. Given that the data did not follow a normal distribution, non-parametric tests were selected; further details of these tests and the rationale for using a non-parametric approach are provided in the Supplementary material. For groupwise comparisons, the Wilcoxon rank-sum test was applied, with a significance threshold set to 0.05.

Friedman’s test for repeated measures was used to investigate the consistency of results for the same subject across different time intervals. This was done for two reasons: first, to establish whether the suggested measures were consistent over time, and thus validate their potential as biomarkers; and secondly, to assess whether the results can be treated as repeated measures. In all groups across both the WPC and DBI, the *P-*value was above 0.05, suggesting that this treatment is appropriate. Further details and assessments of the repeated measures are presented alongside Supplementary Tables 13–16.

In addition, the Kruskal–Wallis test was used to further assess the consistency of results across repeats on an individual subject level. Only a single CG individual provided inconsistent results across repeats for the DBI measure, while all were consistent for WPC.

#### Repeated measurements

Alongside the video segments, five sequential measurements of 3 min were compared. To ensure that the timing within the overall 20 min recording was not a confounding factor, the segments were also shuffled. A randomly selected segment from each subject was chosen, and a rank-sum test was used to assess group differences. This process was repeated 1000 times, and the percentage of tests yielding significant outcomes (*P* < 0.05) was calculated.

#### Effect size

Cohen’s *d* was used to evaluate the effect sizes when comparing the groups.^72^ Further detail is provided in the Supplementary material and Supplementary Tables 1–4. In general, an effect size of *d* = 0.5 is considered medium, while *d* = 0.8 is considered large.

### Classification

Following the calculation of coherence and couplings, classification analysis was performed. A J48 tree classifier machine-learning algorithm was used in WEKA.^73^ Leave-one-out cross validation was performed. Details of the classification analysis are presented in the Supplementary material.

## Results

The Blackpool and HBN data were analysed separately due to procedural differences during data collection. In the Blackpool data, no significant differences were found in the power between groups in the frontal region, over the medium-frequency band (3.5–12 Hz), across any of the time-segments analysed (Supplementary material Sect. 5.1). However, both the amount of time during which regions remained coupled, and the functional connectivity, were reduced in the ASD group. First, we will focus on the wavelet phase coherence results.

### Coherence

**Figure 3:**
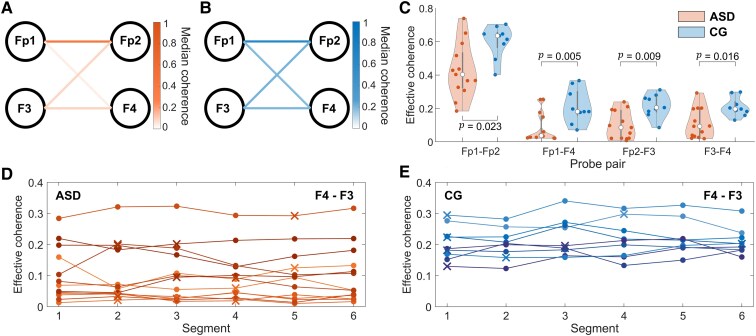
**Coherence results across segments.** (**A**) Heatmap showing the group median coherence in the autism spectrum disorder group (ASD, *N* = 13, male), averaged across theta and alpha bands between probes during the video segment. (**B**) Equivalent heatmap for the control group (CG, *N* = 9, male). (**C**) Median effective coherence in the frontal network evaluated across the medium-frequency band (3.5–12 Hz) for the video segment. Cool coloured (blue) violins represent CG while warm colours (orange) indicates ASD. The median of each distribution is indicated by the central circle, and the box indicates the interquartile range. Each filled datapoint represents the mean coherence across the theta and alpha bands for each participant. The Wilcoxon rank-sum test was used to evaluate group differences (*N* = 13 ASD, *N* = 9 CG). (**D**, **E**) The effective coherence results between F3 and F4 for the sequential, and video segments, in the ASD and CG groups, respectively. Filled circles indicate that the segment was chosen sequentially, while a cross represents a video acquired segment. Each line represents a different participant across repeats (*N* = 13 ASD, *N* = 9 CG for each segment).

Initially considering the video segments in the Blackpool data, functional connectivity is significantly (*P* < 0.05) decreased across all frontal probe pairs in the ASD group. Figure 3A and B shows the group median coherence for the video acquired data in the ASD and CG cases, respectively. Figure 3C illustrates the distribution of the data. Probe pair Fp1–F4 showed the greatest difference between groups (*P* = 0.005).

To assess repeatability in data more prone to movement artefacts, the analysis was performed again in five chronologically selected intervals, each of length 3 min. Figure 3D and E shows the median coherence for each subject across the six segments analysed. The differences between groups in each of these additional segments proved to be significant. In addition, the F3–Fp2 connection was significant at the Bonferroni adjusted *P*-value of 0.0125 for all six intervals initially analysed. The F3–Fp2 connection also had the largest effect size (1.55) of the video segments. Further details regarding the analysis of each segment, including group median values, their corresponding violin plots and all effect sizes are given in the Supplementary material. The level of significance between groups at each frontal probe combination and across all segments is illustrated by the *P*-values in Table 3.

**Table 3:** Reported *P*-values in the video segments for the WPC (top) and DBI (bottom)

	*P*-values						Shuffled repeats (%)
	1	2	3	4	5	Video	
WPC							
Fp1–Fp2	**0.038**	**0.027**	**0.004**	**0.027**	**0.016**	**0.023**	**92**
F3–Fp2	**0.009**	**0.002**	**0.006**	**0.004**	**0.004**	**0.009**	**100**
Fp1–F4	**0.009**	**0.011**	**0.007**	**0.003**	**0.009**	**0.005**	**100**
F3–F4	**0.001**	**0.004**	**0.003**	**0.004**	**0.005**	**0.016**	**100**
DBI							
Fp1 → Fp2	0.081	**0.020**	**0.027**	**0.026**	**0.017**	**0.010**	**72**
Fp1 ← Fp2	**0.047**	**0.003**	**0.031**	**0.006**	**0.014**	**0.004**	**84**
Fp1 → F4	0.108	**0.001**	**0.030**	0.094	**0.032**	**0.003**	**72**
Fp1 ← F4	**0.045**	**0.004**	**0.032**	**0.013**	**0.004**	**0.028**	**92**
F3 → F4	**0.041**	**0.013**	0.123	0.095	**0.017**	0.344	**64**
F3 ← F4	0.070	**0.003**	**0.021**	**0.032**	0.131	**0.031**	**60**
F3 → Fp2	**0.014**	**0.004**	0.065	**0.011**	**0.048**	**0.007**	**76**
F3 ← Fp2	0.071	**0.001**	**0.008**	**0.003**	**0.003**	**0.004**	**96**

Bold numbers indicate statistical significance (*P* < 0.05, *N* = 13 ASD, *N* = 9 CG) obtained using the Wilcoxon rank-sum test. A hyphen between probes indicates WPC was used, while an arrow indicates DBI, with the direction specified. In the WPC group, 24 out of a potential 24 group comparisons proved to be significant. In DBI, 39 out of a possible 48 were significant. The final row indicates the percentage of total temporally shuffled datasets that were significant, for 1000 shuffles of the five sequential segments.

**Figure 4:**
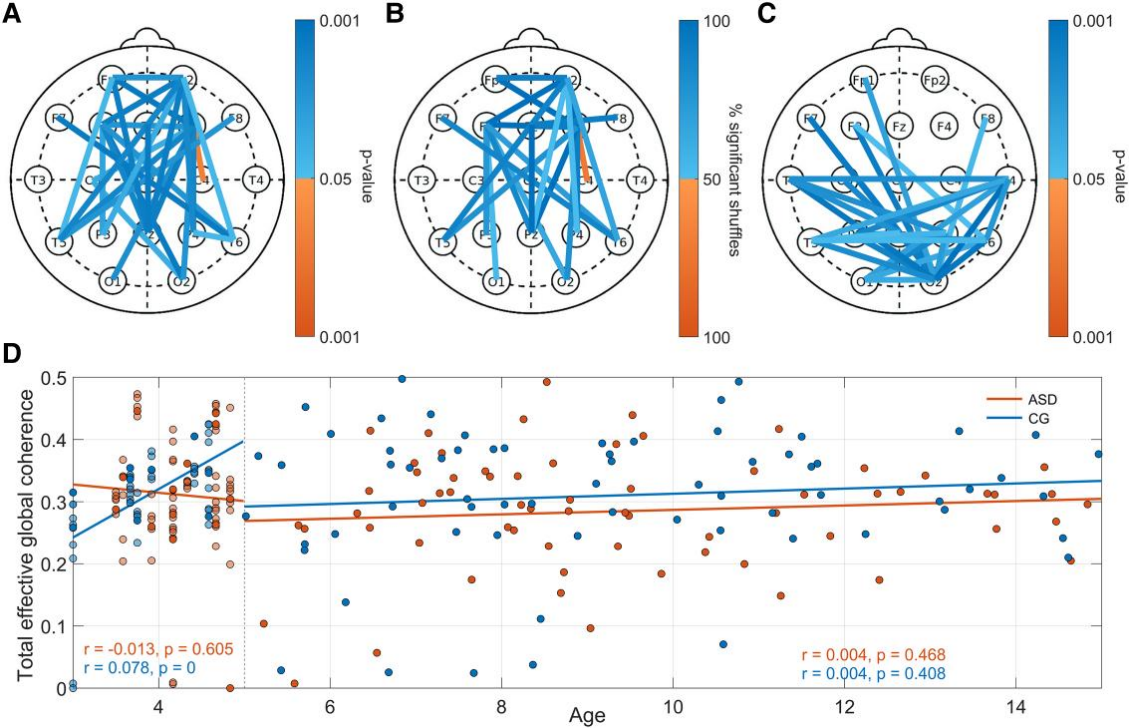
**Coherence differences between autism spectrum disorder (ASD) and control (CG) groups.** (**A**) Coherence differences between probes (3–5-year-old, male, ASD: *N* = 13, CG: *N* = 9). Probe pairs with significant differences (*P* < 0.05, Wilcoxon rank-sum test) are plotted: cool coloured (blue) lines indicate higher coherence in the CG, and warmer coloured (orange) lines indicate higher coherence in the ASD group (**B**) Headmap of significant (*P* < 0.05, Wilcoxon rank-sum test) coherence differences from 1000 randomly shuffled repeats for the 3–5-year-old male group. The headmap shows the percentage of the 1000 tests yielding significant differences. (**C**) Coherence differences for 5–15-year-old males (ASD: *N* = 67, CG: *N* = 66), using the same method as in (**A**). (**D**) Total global effective coherence in the 3.5–12 Hz frequency band for males with and without ASD in both the Blackpool (*N* = 65 ASD, *N* = 45 CG) and Healthy Brain Network (*N* = 67 ASD, *N* = 66 CG) datasets. Each coloured datapoint represents the global effective coherence across the theta and alpha bands for each participant. Regression analysis was performed separately for the two datasets. In the Blackpool data for the ASD group, r = −0.013, *P* = 0.605 and for the CG group r = 0.078, *P* < 0.000. In the HBN data for the ASD group, r = 0.004, *P* = 0.468 and for the CG group r = 0.004, *P* = 0.408. The data were also pooled together. In this case, for the ASD group, r = −0.003, *P* = 0.355 and for the CG group r = 0.003, *P* = 0.305.

To ensure that the time at which the recording was taken was not a confounding factor, shuffled repeats were assessed. All connections for which over half the repeats demonstrate significance are plotted in Fig. 4B. The most consistent differences between groups were found in the frontal network, with 100% of shuffled repeats significantly greater for F3–Fp2, Fp1–F4 and F3–F4 and 92% for Fp1–Fp2.

For the video acquired segment, functional connectivity across the entire head (3.5–12 Hz) was also evaluated, 28 connections showed significantly increased coherence in the controls relative to the ASD group (Fig. 4A). WPC was also assessed in the HBN data (Fig. 4C): 28 significant connections were found, with the majority being more posteriorly located than in the Blackpool cohort.

Global synchrony demonstrated no significant differences in the 3–5 (Blackpool) or the 5–15-year-old (HBN) age ranges between the ASD and CG groups (Fig. 4D). Regression found no significant association with age in the 5–15 range for either the ASD or CG groups.

### Couplings

**Figure 5:**
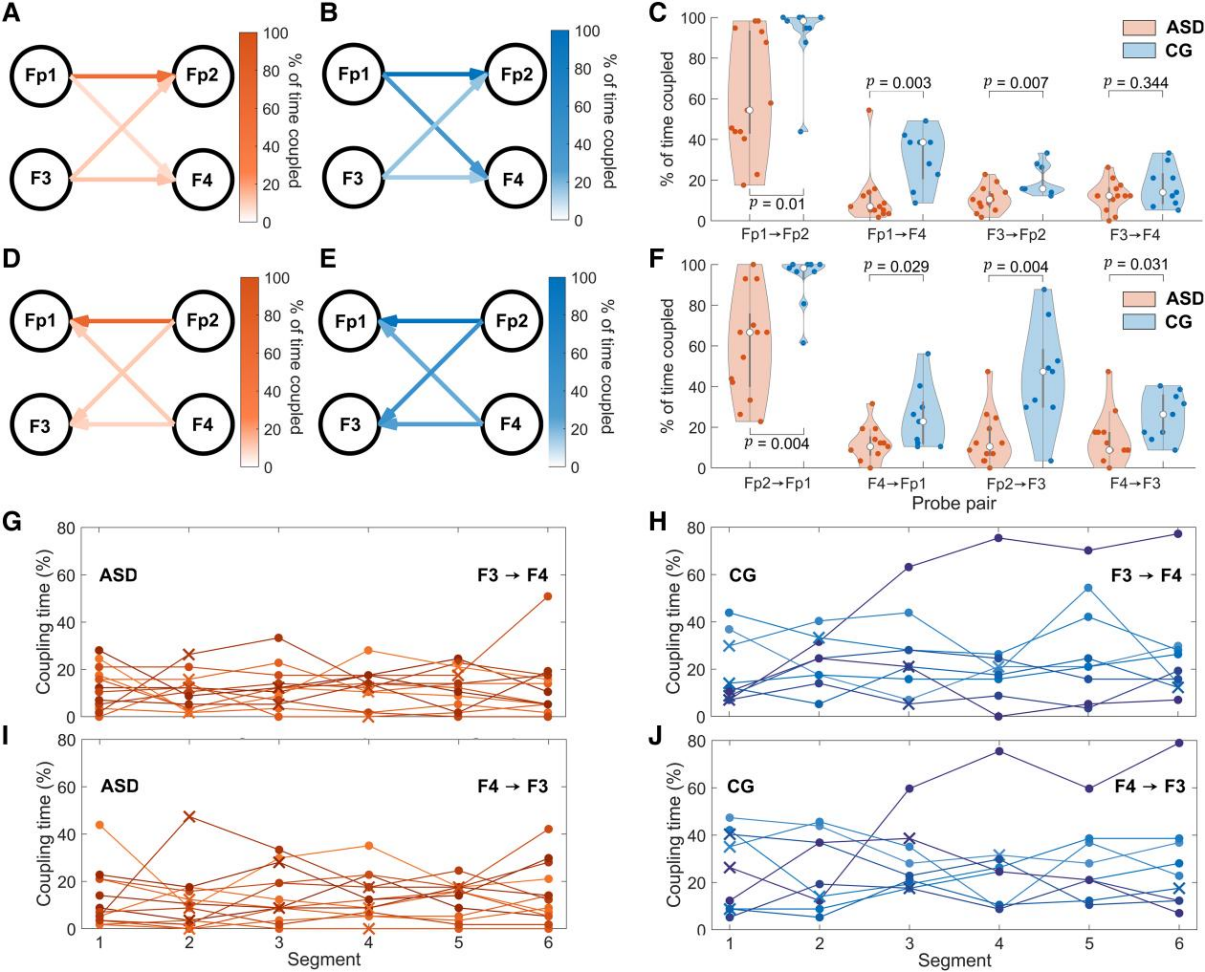
**Coupling time results across segments.** (**A**, **B**, **D**, **E**) Headmaps representing the group median percentage coupling time between probes for the video segment in the autism spectrum disorder group (ASD, *N* = 13, male, warm colours - orange) and the control group (CG, *N* = 9, male, cool colours - blue). (**C**, **F**) Percentage time coupled between the measured EEG signal locations evaluated across the medium-frequency band (3.5–12 Hz) in the video segments, from the left to right (**C**), and right to left (**F**) hemisphere. The Wilcoxon rank-sum test was used to assess group differences (*N* = 13 ASD, *N* = 9 CG). Cool coloured (blue) violins represent the CG while warm colours (orange) indicates the ASD group. The Wilcoxon rank-sum test was used to assess group differences. (**G–J**) The coupling time for each of the sequential, and video, segments. Each line represents a different participant across repeats (*N* = 13 ASD, *N* = 9 CG for each segment). Filled circles indicate that the segment was chosen sequentially, while crosses represent video acquired segments. In this case, the bidirectional coupling between F3 and F4 is illustrated, for the ASD (**G**, **I**) and (**H**, **J**) cases.

Effective connectivity is reduced in the ASD group. Figure 5 illustrates these differences, separated into connections from the left to right hemisphere (Fig. 5A–C) and right to left (Fig. 5D–F) for frontal areas. Each of the probe combinations demonstrated a significantly reduced (*P* < 0.05) coupling time in the video acquired segment, except for F3 → F4. The violin plots of Fig. 5C and F present the distribution of these values, alongside the *P*-value for each bidirectional coupling.

The analysis was repeated across a series of chronologically derived segments. The violin plots representing the distribution of coupling times found for these intervals are illustrated in Supplementary Figs 11–15. Only the couplings Fp2 → Fp1 and F4 → Fp1 were significantly reduced across all six of the intervals under consideration. The *P*-values for each bidirectional probe combination and across all segments are illustrated in Table 3, and Fig. 5A, B, D, and E shows the median time coupled in the video segment for the ASD and CG groups, respectively. Although still relatively high, the percentage of shuffled repeats that were significant when applying DBI was lower than in the WPC case (Table 3). Interestingly, the most repeatable difference between groups in terms of coupling was Fp2 → Fp3, with 96% of shuffled repeats demonstrating significance, consistent with the WPC analysis for Fp2–Fp3. The percentage of time coupled also exhibited more variability across repeats than WPC, as demonstrated by comparing Figs 3D and E and 5G–J. The Kruskal–Wallis test also revealed that one of the CG participants yielded DBI results that were not consistent across time.

### Classification

Using the J48 decision tree algorithm in WEKA, 86% accuracy was achieved when discriminating between ASD and CG in the Blackpool video segment (3–5 years, *N* = 13 ASD, *N* = 9 CG), and 80% was achieved in the older group of the HBN dataset (9–15 years, *N* = 31 ASD, *N* = 33 CG). Further details, including the parameters used as attributes, are provided in the Supplementary material.

## Discussion

The results confirm our hypothesis of reduced connectivity in the alpha and theta bands for young males with ASD, possibly due to the couplings between these areas being more transient.

The time-resolved analysis methods used here are based on the theory of chronotaxic,^74^ finite-time dynamics^75^ and the definition of instantaneous phases.^76^ This approach enables the simultaneous detection of multiple deterministic, nonlinear, multiscale and time-varying interactions that may be neglected by traditional analysis approaches^25,28,29,31-33,77^ or treated as stochastic.^30^

### Functional connectivity

The functional connectivity was assessed using phase coherence. The ASD group was shown to have significantly reduced MF connectivity. This result was replicated across each of the sequential time intervals and also in the HBN data. The largest difference between groups in the Blackpool data was F3–Fp2, which proved to be significant at the Bonferroni adjusted *P*-value of 0.0125 for all six intervals, and 100% of the shuffled repeats. In the HBN data, posteriorly located probe combinations demonstrated more significant connectivity differences between groups. This is likely due to eyes-closed being used predominantly as the HBN protocol, which is known to generate greater occipital alpha band activity.^78^ In addition, the action of opening and closing the eyes periodically may have induced movement artefacts that confounded the connectivity results between frontal probes.

Reduced frontal connectivity in ASD, as found in the Blackpool cohort, confirms the results of some earlier studies,^16,19,21,47,49^ and may predict subsequent outcomes in children as young as three months old.^20^ The consistency of this measure over time probably depends on using a sufficiently large measurement window to capture the underlying oscillatory dynamics. Longer windows ensure the capture of time-varying behaviour across many cycles of the oscillation of interest.

Global coherence demonstrated no significant relationship with age in either the 5–15-year-old (HBN) group or the 3–5-year-old (Blackpool) group. Local coherence differences were much more significant and are promising as a potential determinant. For both groups, the local functional connectivity, evaluated with phase coherence, indicated a consistent trend towards reduced functional connectivity in ASD.

Despite the strength of this result, it is not possible to infer whether it is due to mutual interaction between oscillators, rather than to a shared common influence.

### Effective connectivity

To assess effective connectivity, the couplings were calculated using DBI, which was applied to the Blackpool data. By considering the time over which couplings are significant, another temporal dimension is introduced. Using this framework, it was found that the frontal couplings were present for shorter times in the ASD group than in the neurotypical controls. This reduction in coupling time for ASD individuals may be symptomatic of a reduction in executive function.^21,38-40^ In each frontal probe combination, a greater proportion of the shuffled repeats were significant for WPC than for DBI, indicating that DBI, although able to reveal more information about the dynamics, may be less repeatable than WPC. ASD has been associated with longer dwell times in a disconnected state, and our results support this conclusion for the frontal region.^3^ DBI was not assessed in the HBN data as the changing measurement condition made it inappropriate for evaluation with the Bayesian framework.

Recent publications have emphasized the need to shift the research focus in ASD from functional connectivity analyses to directional, effective connectivity.^1^ Notably, temporal dysregulation has also been identified as a significant factor in ASD.^3,4^ We have presented a method for evaluating the duration of coupling between various brain regions. While we specifically demonstrate the application of this approach in individuals with ASD and within the frontal network, it also holds potential for broader implementation across different cohorts, frequency bands and brain regions.

### Resilience of the phase-based approach

Coherence was reduced in the ASD group for all time intervals investigated across the frontal region. A high level of consistency was found with both methods and for both datasets, despite the increased presence of movement artefacts across the sequentially obtained segments, compared to the video segments. WPC was more reproducible than DBI. Effective clinical implementations of EEG analysis, particularly in paediatric cases, require methods capable of detecting interactions, even in the presence of amplitude perturbations. Evidence regarding connectivity differences in the literature remains inconsistent, with both hyperconnectivity^16,19,21,47,49^ and hypoconnectivity^16,19-21^ being reported in individuals with ASD. These inconsistencies may be due in part to amplitude-weighted measures affecting the reliability of the results.^26^ It is hoped that phase-based approaches will ameliorate this situation.

### Assessing connectivity evaluation methods

Direct comparison between the different methods of calculating connectivity from EEG data is challenging due to the variety of measurement protocols used. Factors such as participant age, sex and state during recording have been shown to influence EEG connectivity.^79-81^ For example, reduced frontal theta/alpha connectivity has been reported^82^ in ASD; however, this finding was based on data collected under eyes-closed conditions, using 2s epochs. The Blackpool data in the present study consisted of 3 min, eyes open EEG recordings, and so one would not necessarily expect identical results. A future methodological project to benchmark connectivity methods against one another, using the ‘same’ dataset in each case, would provide clearer insights into their respective strengths and weaknesses. We emphasize, however, that the methods used in the present study amount to much more than just another alternative approach to EEG analysis. As discussed in the Introduction, they are the first to be applied that are able to take fully into account the inherent non-autonomicity of biological oscillators. The advantages and, in many cases, necessity of using such an approach are explored and described in detail in the papers cited.^30,36,83^ It is thus to be expected that the approach used here should yield more insightful results than any of the alternatives.

### Feasibility of biomarkers

Given the lack of consensus in the literature, novel analytical approaches may play a crucial role in elucidating the underlying connectivity differences present in ASD. The brain is an incredibly complex organ, and despite a multitude of research into biomarkers for ASD assessment, a single diagnostic test is still beyond reach. Here, by explicit consideration of time as a physical parameter during data analysis and consideration of finite-time dynamics, we have paved a way for novel biomarkers.

Due to the demand for fast throughput in clinical settings, 20 min EEG recordings may not always be achievable. Additionally, when measuring brain activity in children, movement artefacts are inevitable and should be anticipated and accounted for. It is therefore crucial to use methods that yield reproduceable findings despite the presence of movement artefacts.

Like other dynamical analyses of physiological data, EEG investigations are complicated by the thermodynamically open nature of biological systems and their inherent fluctuations. The underlying oscillatory processes are consequently non-autonomous, exhibiting characteristic frequencies that vary in time. This feature must be considered in the data analysis^36,83^ for the results to be reliable. Explicit consideration of recording length is paramount when assessing oscillatory neural activity, especially when considering interactions.

The vast heterogeneity of ASD means that individuals with the condition are highly unlikely to share identical EEG signatures. A range of biomarkers, each with their own behavioural correlate, may enable a more stringent evaluation. Further research considering the putative relationship between frontal connectivity and restricted and repetitive behaviours in ASD across various age ranges, using larger cohorts and comparing with behavioural measures may elucidate the potential behavioural correlates suggested by this investigation. Using a very simple classification method, a classification accuracy of 86% was obtained.

In our current approach, we employ the same frequency band for both probes in the DBI calculation. However, alternative cross-frequency approaches can also be explored. For example, one could investigate the influence of delta activity in one region on alpha activity in another region.^84^ Cross-frequency coupling functions have demonstrated aberrations in various neurological conditions^85^ and may offer an additional avenue for assessing ASD.

In isolation, the functional connectivity results may indicate that there is simply a lower, constant, coherence across time. The coupling results supplement the WPC by showing that the amount of time over which coupling is present is also reduced in ASD. By unlocking the temporal dimension in this way, additional information about the dynamical nature of the couplings is revealed. In combination, both the connectivity strength, and the time over which the regions are coupled, are shown to be significantly different in ASD, and may provide the basis for a potential biomarker.

### Limitations and strengths

Although we report the potential of frontal network connectivity as a biomarker for ASD, more research based on larger groups and different age bands would be required to ascertain if this outcome can be realized. Additional analyses with the same age range and measurement procedure are needed to validate the findings.

It is crucial to acknowledge that a comprehensive diagnostic tool would necessitate multiple markers, each capturing distinct aspects of the ASD experience. One may argue that the observed decrease in frontal connectivity in the Blackpool data may be associated with the characteristic restricted and repetitive behaviours exhibited by some individuals with ASD.^38,45,86^ However, using the HBN data, we show that phase coherence is also a useful marker when the measurements are conducted in a different brain state. Further investigations could elucidate the origins of other behavioural correlates, such as social and communication difficulties.

The size of the Blackpool data is relatively small. Several arguments indicate, however, that the conclusions are reliable. First, as shown in the results section and the Supplementary material, the effect size is large. Secondly, the Blackpool recordings were sufficiently long to allow for six different 3 min segments to be analysed, and thus for the reproducibility of the results to be evaluated. The results ensured that the inferred interactions were not spurious. The additional HBN dataset, coming from a larger cohort with a wider age range, provided a further reassuring validation of our conclusions.

Only boys were recruited in the present study as the neural signatures of ASD are known to vary between the sexes^52,80^ and a sufficient number of females could not be recruited within the time frame of the study to warrant an additional investigation.

The effect of volume conduction may have influenced the effect sizes of the phase coherence and couplings analyses. In the absence of reliable models to take volume conduction into account, we have mitigated this problem in several ways. It is known that the spatial separation minimizes the impact of volume conduction.^87^ The probe density in both datasets is relatively small, hence even the nearest probes can be expected to be weakly affected. Next, the probe pair F3–Fp2, which are widely separated within the frontal network, exhibited the most pronounced differences in coherence between groups. Furthermore, as our primary aim is to compare coherence and coupling values between ASD and neurotypical control groups, it is reasonable to assume that volume conduction would likely affect both groups similarly.

The use of phase coherence and coupling functions allow an investigation of neural dynamics with reduced influence from the movement artefacts and noise that can bedevil amplitude-based methods. The robustness to amplitude effects enables the analysis of relatively long segments that contain more information than the shorter epochs often investigated in EEG studies.^15,18,19,79,88^

## Concluding remarks

Despite the condition having been recognized for over a century, ASD diagnoses still depend on behavioural tests and interviews. As well as being time-consuming, these assessments require certain characteristic features to be apparent, meaning that most children do not receive a diagnosis until age 3/4 or even later.^89^ A diagnostic tool that revealed the presence of ASD before its behavioural emergence could be useful, quite apart from its utility in assessing the response to intervention. Our investigation of electrophysiological signatures in ASD and neurotypical children provides a promising step towards putative biomarkers for identifying and categorizing the condition.

## Supplementary Material

fcaf084_Supplementary_Data

## Data Availability

The EEG data recorded in Blackpool, codes used in the work and some additional figures in .mat format are publicly available on Lancaster University’s Pure database, DOI: 10.17635/lancaster/researchdata/694. The phenotypic data from the HBN may be accessed upon request from the biobank and following the acceptance of a data usage agreement. The majority of the analyses were done using the MODA (Multiscale Oscillatory Dynamics Analysis) software toolbox, which is publicly available at https://github.com/luphysics/MODA, and the background of the methods is provided in its handbook.^90^
