# Supplementary material for: Theta and alpha connectivity in children with autism spectrum disorder
Source: Brain Commun. 2025 Feb 19;7(2):fcaf084. doi: 10.1093/braincomms/fcaf084 (PMC11894932; doi:10.1093/braincomms/fcaf084)
Supplement: fcaf084_Supplementary_Data [file fcaf084_supplementary_data.pdf]

SUPPLEMENTARY MATERIAL
for
Theta and alpha connectivity in children with autism spectrum disorder

Samuel J.K. Barnes[1] Megan Thomas[2,3] Peter V.E. McClintock[1] Aneta Stefanovska[1*]

[1]Lancaster University Physics Department, Lancaster, LA1 4YB, England
[2]Department of Pædiatrics, Blackpool Teaching Hospitals NHS Foundation Trust, Blackpool, United Kingdom
[3]Now at Department of Pediatrics, Faculty of Medicine, Dalhousie University, Halifax, Nova Scotia, Canada
*Corresponding author: aneta@lancaster.ac.uk

Contents

1 Introduction **2**

2 Model procedure and parameters **2**
2.1 Wavelet phase coherence (WPC) model . . . 2
2.2 Dynamical Bayesian inference (DBI) Model . . . 2

3 Signal preprocessing **3**

4 Movement artefacts and amplitude weighting **3**

5 Statistical tests **4**
5.1 Wilcoxon rank-sum test . . . 4
5.2 Friedman's test for repeated measures . . . 5

6 Effect sizes **5**
6.1 Study sensitivity . . . 5
6.2 Wavelet phase coherence effect size . . . 5
6.3 Dynamical Bayesian inference effect size . . . 7

7 Controlling for relaxation aids in resting state measurements **8**

8 Classification **11**

9 Additional results **11**
9.1 Power . . . 11
9.2 Wavelet phase coherence . . . 12
9.3 Dynamical Bayesian inference . . . 15

10 Healthy Brain Network **21**
10.1 Inclusion criteria . . . 21
10.2 Splitting the age groups . . . 22

1 Introduction

This document provides Supplementary Material for "Frontal connectivity in children with autism spectrum disorder". The procedures and parameters used to generate illustrative models in the paper are given in section 2. Section 3 describes the preprocessing that was performed on the measured EEG signals. An example using real data to illustrate why phase, rather than amplitude-weighted, measures were chosen for the investigation is provided in section 4. In section 5, the statistical methods used, and the reasoning behind their application, are described. This includes the group medians and Friedman's test results, which confirm that there is consistency across the repeats for each person. Additional details pertaining to the effect sizes are are given in section 6. Section 7 demonstrates the results when controlling for relaxation aids in the resting state. The classification analyses performed are described in section 8. Following this, section 9 provides violin plots for each of the repeats to highlight the distribution of the data across all of the segments in the Blackpool results. Finally, section 10. gives further analysis of the validation dataset.

2 Model procedure and parameters

Two models were employed to demonstrate the methods used in the paper; they relate to Figs. 1. and 2. of the main text.

2.1 Wavelet phase coherence (WPC) model

Wavelet phase coherence assesses the degree to which a pair of oscillatory processes share a common phase evolution. To demonstrate cases which display both high and low coherence, a pair of time series were numerically generated.

The first series, $x_1(t)$ contains two time-varying oscillatory modes centred at frequencies $\omega_1 = 5$Hz and $\omega_2 = 9.5$Hz. The modulation frequency of these modes is $\omega_{m1} = 0.005$Hz and $\omega_{m2} = 0.015$Hz, while the amplitudes of modulation are $A_1 = 2$, $A_2 = 8$. The following differential equation for the phases of the system is solved using the fourth-order Runge-Kutta approach (RK4). A total time of 400 seconds was iterated over, with a time step of 0.005s for each oscillatory mode to determine the phases, α_i, present in the system at each time.

$$\dot{\alpha}_i = \omega_{0i}(t) = \omega_i + A_i \sin(\omega_{mi} t) \tag{1}$$

These derived phases are then used to generate the signal with two independent modes, such that,

$$x(t) = 5\cos(\alpha_1 t) + 5\cos(\alpha_2 t) + \eta(t), \tag{2}$$

where η is an additive pink $(\frac{1}{f})$ noise term. The noise was obtained using the Matlab built-in *pinknoise* function, with the output being multiplied by a factor of 150.

The above enables the user to generate a signal with two oscillatory modes. To enable the juxtaposition of coherence both with and without a constant phase difference, a further time series is needed. As such, an additional time series was generated, which lacked the high frequency oscillatory mode for the first 200s. This initial series was subsequently concatenated with another 200s time series, simulated with both modes. This concatenated time series is represented by the orange lines in Fig.1A, B of the main text. and is also represented in the time/frequency domain (Fig.1C main text), while its time-averaged power is given in Fig.1D (main text). The phases of this signal are also represented over a short interval in the orange lines of Figs. 1E, F in the main text.

To evaluate the time/frequency representation of Fig. 1C, a Morlet wavelet was used with a frequency resolution parameter of $F_0 = 4$. The wavelet transforms of both the aforementioned signals served as inputs to the coherence calculation.

2.2 Dynamical Bayesian inference (DBI) Model

While the WPC model as outlined above only needed to demonstrate the presence of oscillatory modes, the DBI model needed to illustrate a coupling between time series. A total period of 1000 seconds were simulated across with a time step of 0.03s using RK4 to find the phases of a pair of unidirectionally coupled phase oscillators. The frequencies of these modes do not vary in time and $\omega_1 = 6$Hz, $\omega_2 = 9$Hz,

$$\dot{\phi}_1 = \omega_1 + \eta(t),$$
$$\dot{\phi}_2 = \omega_2 + q_2(\phi_1, \phi_2) = \omega_2 + E\cos(\phi_1 + \pi/2.5) + \eta(t). \tag{3}$$

Where the noise η, in this case, is white Gaussian and applied to the instantaneous frequency, implemented using the built-in *wgn* Matlab function. In the above example, E, is the coupling strength between the oscillators and can be varied to increase or decrease the amount of information transfer between the systems. This is shown in Fig. 2 of the main text.

Once this ground truth had been established, the couplings were verified using dynamical Bayesian inference. The parameters applied for the DBI are as follows: window size = 100s, propagation constant = 0.2 and the overlap parameter = 1 (no overlap between windows).

3 Signal preprocessing

During the collection of the Blackpool data, two different reference electrode positions were used. To account for the resultant bias in recording, the data were re-referenced to the common average. This involved subtracting the average sum of all 19 electrodes from each individual probe's time series. The equations describing this procedure are:

$$V_{CA} = \frac{1}{N}\sum_{i}^{N} V_i, \tag{4}$$

$$V_i' = V_i - V_{CA}, \tag{5}$$

where V_{CA} is the common average, V_i is the time series for the individual probes, V_i' is the rereferenced time series, and N is the total number of electrodes ($N = 19$). After the time series were rereferenced, they were bandpassed with a Butterworth filter of order proportional to the frequency band of interest, and then detrended by subtracting the 3rd order polynomial. This was done using the MATLAB functions bandpass and detrend respectively.

4 Movement artefacts and amplitude weighting

The methods used throughout this investigation are independent of amplitude and instead, focus solely on phase dynamics. Alternative measures that also incorporate amplitude information are frequently employed to evaluate functional connectivity. Among these measures, one of the most extensively utilised is amplitude-weighted phase coherence (AWPC) [6, 7]. This method considers both the amplitude and phase at each point in the time/frequency domain to calculate coherence. For a pair of time series AWPC is defined as the normalised wavelet cross-spectrum at each point in time and frequency [6],

$$AWPC(t,f) = \frac{|W_{12}(t,f)|}{[W_{11}(t,f)\cdot W_{22}(t,f)]^{1/2}}. \tag{6}$$

Where W_{12} is the wavelet cross-spectrum, and W_{11}, W_{22} are the auto-spectra. As before, a value of 1 is found for perfect coherence, while 0 represents a complete lack of coherence. The key distinction between these approaches is that a simultaneous amplitude change for a pair of time series will cause an increase in the AWPC, but not in the WPC [1, 5]. This makes WPC more resistant to sudden amplitude perturbations – such as those arising from movement artefacts – than AWPC [1].

Data containing a clear movement artefact were analysed to illustrate the differences between these methods. These time series were selected by visual inspection of the large amplitude spike, which was concurrently present across all probes. This spike is likely unrelated to the underlying neural dynamics, and so obfuscates approaches that incorporate amplitude information.

Despite the pervasive effect of the movement artefact on the data, Fig. 1. illustrates that WPC remains resilient as a representation of shared phase information between the signals. In contrast, the amplitude dependence of AWPC suffers a significant susceptibility to simultaneous amplitude changes, exhibiting a peak at around 90 seconds that coincides with the mutual spike. In addition, EEG data are particularly affected by noise and amplitude effects due to, for example, differential signal attenuation

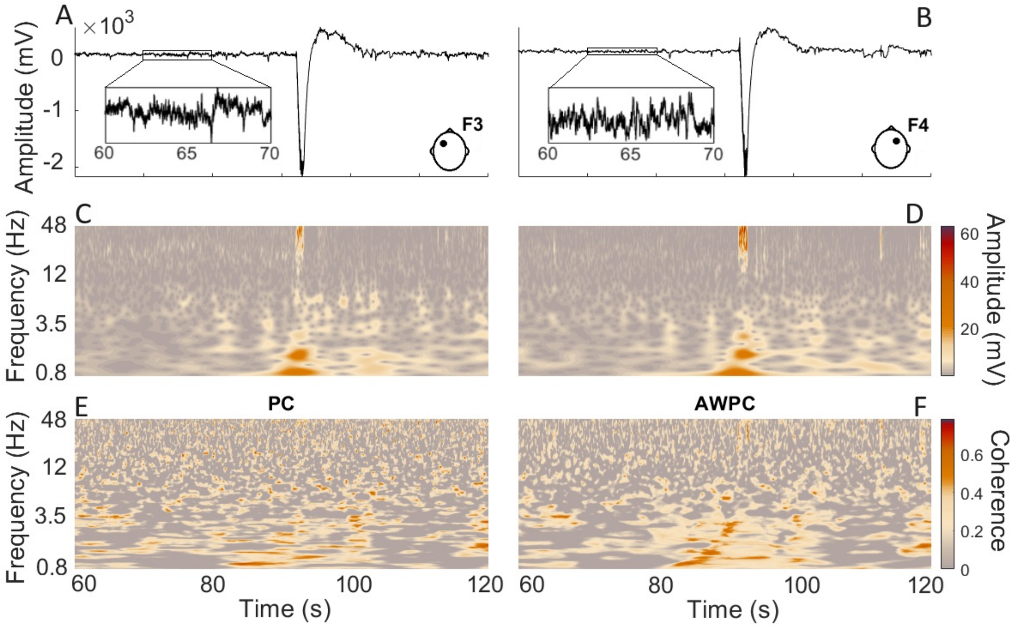

Supplementary Figure 1: Movement artefact present the ASD time series (subject 4, segment 3). A pair of EEG time series from probes F3 (A) and F4 (B), each containing a large artefact. Their respective wavelet transforms (C, D) further indicate a multiscale spike in power. Both wavelet transforms (C, D) serve as inputs for the respective coherence calculations (E, F). The time-localised phase coherence (E) is fairly resilient against this perturbation when compared to its amplitude-weighted counterpart (F). The time-localized coherence was calculated by subtracting the 50th percentile of intersubject surrogates (72 for the control group and 156 for the ASD group). Any coherence values that fell below this threshold were set to zero.

arising from the varying separations of EEG electrodes from the cortical surface. The relative resilience of WPC to amplitude effects suggests that it may provide a more appropriate approach for the investigation of functional connectivity.

A Morlet wavelet with a frequency resolution parameter of $F_0 = 2$ was used. The mean value of 156 intersubject surrogates at each point in the time/frequency domain was used to reduce the amount of spurious time-localised coherence that was detected. Despite this, a larger degree of seemingly coherent behaviour is present in the AWPC compared to the PC, due to the amplitude weighting, which detects a simultaneous spike in both data. Phase coherence is robust against this perturbation.

5 Statistical tests

An assumption of normally distributed data is needed when using parametric approaches; however, the results were generally not normally distributed. Given this lack of Gaussianity, non-parametric tests were applied to compare differences between and within groups.

5.1 Wilcoxon rank-sum test

To assess differences between the groups while acknowledging the non-normal distribution of the data, the Wilcoxon rank-sum test was used. The null hypothesis was that there was no difference in connectivity between the CG and ASD groups.

All of the results from the ASD and CG groups were first ranked. The summation of all the ranks for each group was then calculated. The difference between these sums was then used to assess the statistical significance of any difference between groups. A p-value could then be calculated, and if this fell below 0.05, then we were able to reject the null hypothesis.

5.2 Friedman's test for repeated measures

Friedman's test serves as a non-parametric alternative to the repeated measures ANOVA, aiming to uncover variations among the same subjects over multiple time points. Here, we utilise this test to assess the consistency of results for all subjects across different time intervals, ensuring that results are consistent across repeats. This consistency is vital for two reasons. First, in the context of diagnostic tests, biomarkers must be stable regardless of when a measurement is taken; our analysis seeks to assess this required consistency. Secondly, this approach allows us to see if we can treat the results as repeated measures.

As with the Wilcoxon test outlined above, the data are ranked. In this case, however, they were ranked across the repeats at a given probe pair for each subject. The sum of each column, corresponding to the rank-sum of each repeat, is then taken. These rank-sums are then compared to assess whether there are significant differences at different measurement times.

6 Effect sizes

A post hoc analysis was conducted to determine the effect size using Cohen's d [2]. This metric relies on the means of the two groups being compared, denoted as m_1 and m_2, along with the pooled standard deviation sd which is calculated as

$$sd = \sqrt{\frac{(n_1 - 1)SD_1^2 + (n_2 - 1)SD_2^2}{n_1 + n_2 - 2}}, \tag{7}$$

where n_1 and n_2 represent the sample sizes of the two groups, and SD_1 and SD_2 denote their respective standard deviations. Cohen's d is then computed from the formula:

$$d = \frac{m_1 - m_2}{sd}. \tag{8}$$

In general, an effect size of $d = 0.5$ is considered medium, while $d = 0.8$ is considered large.

6.1 Study sensitivity

The study's sensitivity was assessed using G*Power [3], with a screenshot of the test being presented in Fig. 2. The analysis incorporated a power of 0.5, a significance level of 0.05, and the sample sizes (13 ASD and 9 CG) utilized in the investigation as input parameters.

As depicted in Fig. 2, the exploratory nature of this study resulted in a large effect size of 1.387, due to the modest sample sizes. However, as demonstrated later, many of the repeats surpassed this threshold.

6.2 Wavelet phase coherence effect size

Effect sizes for WPC were initially calculated using the Blackpool dataset. Specifically, the analysis focused on four probe pairs that demonstrated the most significant differences between groups. These probe pairs were evaluated across the six data segments, yielding Cohen's d values ranging from 1.051 to 1.884, with a mean of 1.393. All observed effect sizes in the frontal network exceeded 1 and are shown in Tab. 1.

Supplementary Table 1: Effect sizes for all segments in the inter-hemispheric frontal network evaluated across the medium frequency band (3.5-12 Hz) between groups. The numbered sections 1-5 were found chronologically, while the video segment reflects data intervals selected for their relative lack of movement artefacts.

**From/To**	**Effect sizes**					
	**1**	**2**	**3**	**4**	**5**	**Video**
**Fp1 - Fp2**	1.051	1.295	1.133	1.182	1.191	1.232
**F3 - Fp2**	1.376	1.884	1.476	1.587	1.502	1.554
**Fp1 - F4**	1.273	1.227	1.232	1.630	1.239	1.266
**F3 - F4**	1.357	1.534	1.526	1.883	1.492	1.311

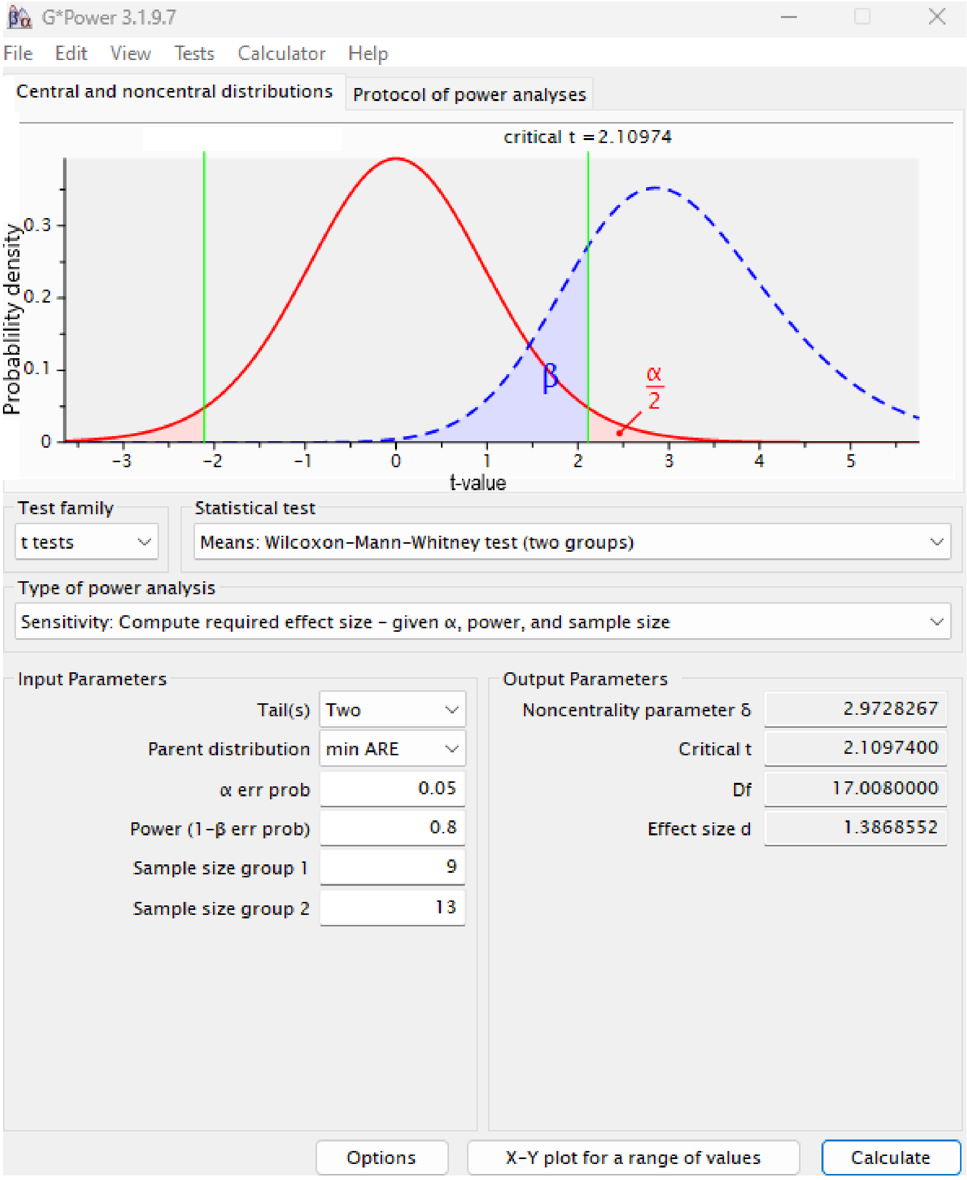

Supplementary Figure 2: Screenshot of the G*power software demonstrating the input and output parameters used. Given an input of 9 control group individuals and 13 participants with autism spectrum disorder and a two tailed distribution. The parent distribution is set to min ARE (minimum asymptotic relative efficiency) to optimise the analysis. The alpha error probability (α err prob) was set to 0.05; this is the significance level of the test. The power, 1–beta error probability (1-β err prob) is the probability of not detecting a true effect and was set to 0.8 (meaning there is an 80 % chance of detecting a true event if one exists). For our purposes, the important output is the effect size, d, which indicates the value that effect sizes must be larger than given the sample sizes and significance level.

Effect sizes from the HBN network data were also calculated. For reasons explained in section 10.2, the entire age group (5-15) and older age group (9-15) were considered separately. Tab 2 presents the effect sizes for each probe pair that exceeded a significance threshold of 0.01 in the older group when applying the Wilcoxon rank-sum test.

Supplementary Table 2: Effect sizes for all segments in the Healthy Brain Network evaluated across the medium frequency band (3.5-12 Hz) for the older (9-15 years-old) group. The probe pairs indicated here all had a p-value less than 0.01 from a Wilcoxon rank-sum test.

**C3-PZ**	**F3-O1**	**F3-O2**	**F3-T3**	**F3-T6**	**O1-P4**	**O2-P3**
0.63	0.70	0.86	0.69	0.79	0.70	0.65

**O2-T3**	**O2-T4**	**P3-P4**	**P-3PZ**	**P3-T4**	**P3-T6**	**T3-T4**
0.78	0.86	0.69	0.65	0.80	0.71	0.75

As elaborated upon in section 10.2, there was more significance in the older group, likely due to their increased ability to tolerate a more demanding measurement protocol. Coherence between probe pair O2-T4 demonstrated the largest effect size (0.86 in the older group). Interestingly however, significance was retained when considering the 5-15 years-old group. Tab. 3 demonstrates the effect sizes in this case.

Supplementary Table 3: Effect sizes for all segments in the Healthy Brain Network evaluated across the medium frequency band (3.5-12 Hz) for the older (5-15 years-old) group. The probe pairs indicated here all had a p-value less than 0.01 from a Wilcoxon rank-sum test.

**F3-O2**	**F7-O2**	**F7-Pz**	**O2-T3**	**O2-T4**	**O2-T6**
0.48	0.44	0.49	0.46	0.62	0.27

6.3 Dynamical Bayesian inference effect size

For the DBI, eight directional probe pairs in the frontal network were evaluated across six time segments. The range of Cohen's d-values spanned from a minimum of 0.554 to a maximum of 2.18, with a mean value of 1.17. These are presented in Tab. 4

Supplementary Table 4: Effect sizes across retests for frontal networks evaluated across the medium frequency band (3.5-12 Hz) between groups. The numbered sections, 1-5, were found chronologically, while the video segment reflects data intervals selected for their relative lack of movement artefacts.

**From/To**	**Effect sizes**					
	**1**	**2**	**3**	**4**	**5**	**Video**
**Fp1 → Fp2**	0.834	1.318	1.312	1.027	1.126	1.165
**Fp1 ← Fp2**	0.690	1.273	1.187	1.344	1.450	1.519
**Fp1 → F4**	0.772	1.664	1.178	0.783	1.081	1.473
**Fp1 ← F4**	1.025	1.507	1.081	1.175	1.326	1.118
**F3 → F4**	1.115	1.170	0.750	0.966	0.852	0.554
**F3 ← F4**	0.817	1.481	1.023	1.068	0.766	0.958
**F3 → Fp2**	1.119	1.654	0.993	1.262	1.033	1.333
**F3 ← Fp2**	0.848	2.178	1.377	1.614	1.542	1.664

Despite the relatively modest sample sizes employed in this study, the observed differences, as indicated by Cohen's d, were fairly large. This also motivates further exploration of the suggested electroencephalographic measures with larger sample sizes to enhance the validity of findings.

7 Controlling for relaxation aids in resting state measurements

In this study, a resting-state paradigm was employed. Some participants occasionally found it difficult to maintain an eyes-open, restful state during measurements. To help them remain relaxed, either bubbles or a smartphone were used as a focus, with these aids used slightly more often in the ASD group than in the control group (46% in ASD vs. 22% in CG). To ensure these interventions did not bias the results, the analysis was repeated after controlling for this factor. Specifically, ASD participants 12, 11, and 10 (who used the screen) and participant 2 (who used bubbles) were excluded from the analysis. This adjustment resulted in 9 participants per group, with no bubbles used in either group and both groups having 22% of participants using the screen in the resting state. The same analysis was performed as in the main manuscript.

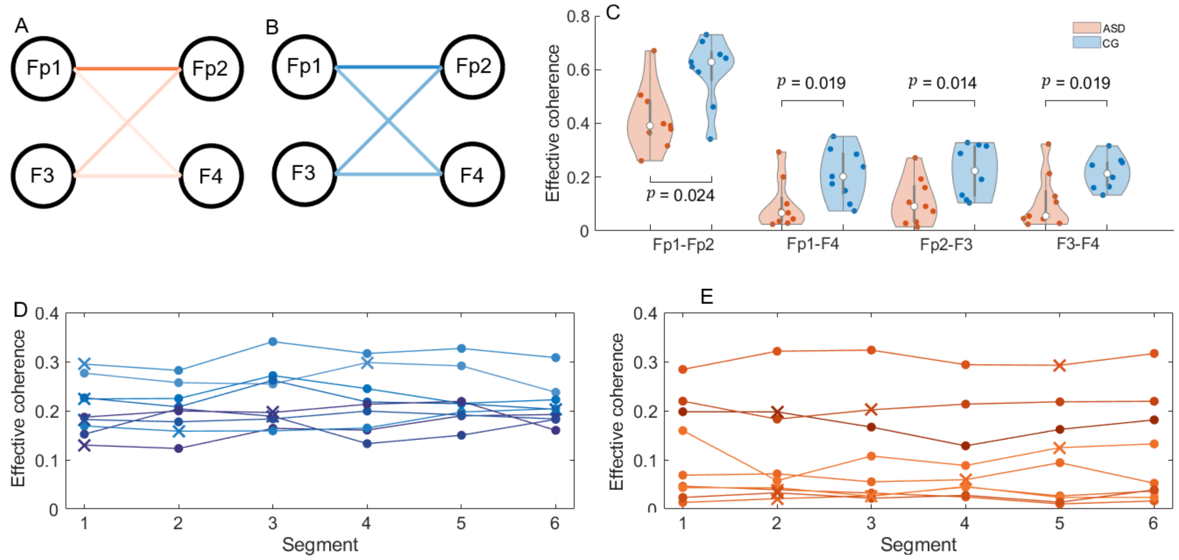

Supplementary Figure 3: Coherence results across all segments when controlling for resting state relaxation aids (A) Headmap representing the group median coherence averaged across the theta and alpha bands following the subtraction of intersubject surrogates between probes for the video segment in the autism spectrum disorder (ASD, $N = 9$, male) group (B) and the control group (CG, $N = 9$, male). (C) Median effective coherence between the measured EEG signal locations evaluated across the medium frequency band (3.5-12 Hz) in the video segment. Blue violins represent CG while red indicates ASD. The median of each distribution is indicated by the white circle and the box indicates the interquartile range. Each coloured datapoint represents the mean coherence across the theta and alpha bands for each participant. The Wilcoxon rank-sum test was used to evaluate the p-values ($N = 9$ ASD, $N = 9$ CG). (D) The effective coherence results for each of the sequential, and video, segments. Filled circles indicate that the segment was chosen sequentially, while a cross represents video acquired segments. Each line represents a different participant across repeats ($N = 9$ ASD, $N = 9$ CG for each segment). In this case, the probe pair F3-F4 is illustrated, for the ASD (D) and CG (E) cases.

The same pattern was observed in the coherence results as had been obtained when using relaxation aids as described in the main manuscript; and the same was true for the dynamical Bayesian inference results (Figs. 3 and 4 respectively). To ensure the results were consistent across the segments investigated, this analysis was repeated.
The same pattern, with significance regardless of the time measurements were conducted, were found when the relaxation states were matched. The slightly lower p-values (Tab. 5 for coherence and Tab. 6 for Bayesian) are likely due to the reduced statistical power as we have decreased the size of the ASD group.

Bayesian results followed the same trend. With slightly lower significance likely due to the reduced sample size, but the same patterns identified. Effect sizes were subsequently checked for both analysis approaches and across the segments (Tab. 7 and 8).
Generally, our results were very similar, indicating that the resting state paradigm made little difference. The small changes to p-values can be explained as the smaller groups hold less statistical strength.

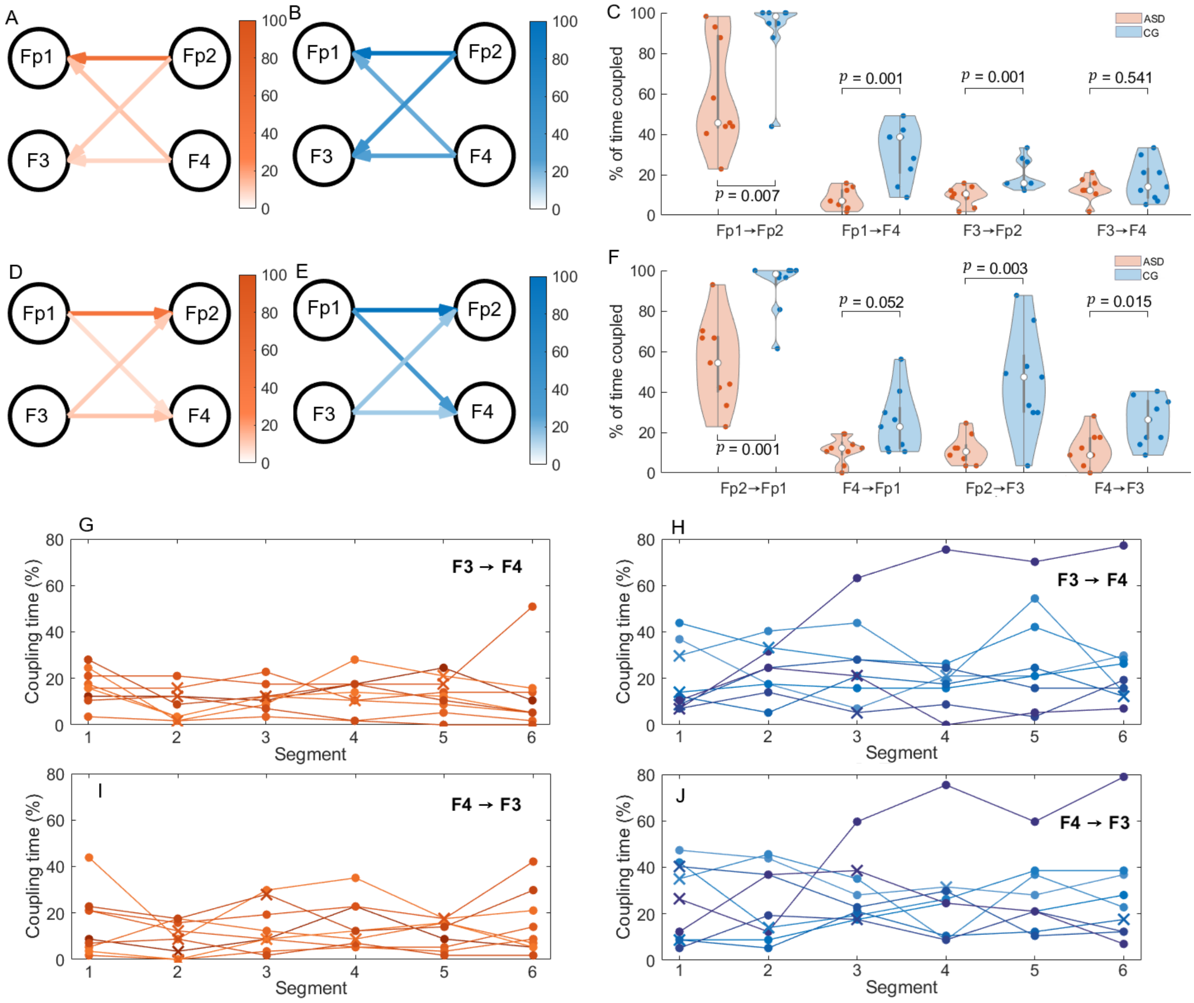

Supplementary Figure 4: Absolute and p-values for the dynamical Bayesian inference coupling time in the groups controlling for resting state. (A, B, D, E) Headmaps representing the group median percentage coupling time between probes for the video segment in the autism spectrum disorder (ASD, $N = 9$, male participants) group (orange) and the control group (CG, $N = 9$, male participants) (blue). The 95th percentile of intersubject surrogates was applied to remove the effect of spurious interactions. (C, F) Percentage time coupled between the measured EEG signal locations evaluated across the medium frequency band (3.5-12 Hz) in the video segments, from the left to right (C), and right to left (F) hemisphere. The Wilcoxon rank-sum test was used to assess group differences ($N = 9$ ASD, $N = 9$ CG). Blue violins represent CG while red indicates ASD. The Wilcoxon rank-sum test was used to evaluate the p-values. (G-J) The coupling time for each of the sequential, and video, segments. Each line represents a different participant across repeats ($N = 9$ ASD, $N = 9$ CG for each segment). Filled circles indicate that the segment was chosen sequentially, while crosses represent video acquired segments. In this case, the bidirectional coupling between F3 and F4 is illustrated, for the ASD (G, I) and (H, J) cases.

Supplementary Table 5: p values for all segments in the inter-hemispheric frontal network evaluated across the medium frequency band (3.5-12 Hz) between groups when the resting state relaxation aids are matched. The numbered sections 1-5 were found chronologically, while the video segment reflects data intervals selected for their relative lack of movement artefacts.

**From/To**	***p*-values**					
	**1**	**2**	**3**	**4**	**5**	**Video**
**Fp1 - Fp2**	**0.024**	**0.014**	**0.024**	**0.014**	**0.006**	**0.008**
**Fp1 - F4**	**0.014**	**0.024**	**0.019**	**0.008**	**0.024**	**0.006**
**F3 - Fp2**	0.050	**0.011**	**0.014**	**0.011**	**0.008**	**0.024**
**F3 - F4**	0.094	**0.011**	**0.019**	**0.024**	**0.031**	**0.04**

Supplementary Table 6: p-values across retests for frontal networks evaluated across the medium frequency band (3.5-12 Hz) between groups when the resting states are matched. The numbered sections, 1-5, were found chronologically, while the video segment reflects data intervals selected for their relative lack of movement artefacts.

**From/To**	***p*-values**					
	**1**	**2**	**3**	**4**	**5**	**Video**
**Fp1 → Fp2**	0.052	**0.012**	**0.008**	**0.012**	**0.011**	**0.007**
**Fp1 ← Fp2**	0.057	**0.001**	**0.020**	**0.006**	**0.013**	**0.001**
**Fp1 → F4**	0.098	**0.0002**	**0.029**	**0.037**	**0.023**	**0.001**
**Fp1 ← F4**	0.072	**0.001**	0.065	**0.009**	**0.013**	0.052
**F3 → Fp2**	0.095	**0.004**	0.141	**0.042**	0.141	**0.001**
**F3 ← Fp2**	0.098	**0.0004**	**0.020**	**0.004**	**0.005**	**0.003**
**F3 → F4**	0.248	**0.007**	0.197	0.183	**0.013**	0.541
**F3 ← F4**	0.0.197	**0.001**	0.052	**0.037**	0.141	**0.015**

Supplementary Table 7: Median effect sizes for all segments in the inter-hemispheric frontal network evaluated across the medium frequency band (3.5-12 Hz) between groups when the resting states are matched. The numbered sections 1-5 were found chronologically, while the video segment reflects data intervals selected for their relative lack of movement artefacts.

**From/To**	**Effect sizes**					
	**1**	**2**	**3**	**4**	**5**	**Video**
**Fp1 - Fp2**	1.283	1.643	1.430	1.505	1.539	1.679
**F3 - Fp2**	1.116	1.624	1.335	1.440	1.392	1.490
**Fp1 - F4**	1.198	1.147	1.132	1.405	1.069	1.287
**F3 - F4**	1.096	1.408	1.366	1.651	1.249	1.254

Supplementary Table 8: Dynamical Bayesian inference effect sizes across retests for frontal networks evaluated across the medium frequency band (3.5-12 Hz) between groups when the resting states are matched. The numbered sections, 1-5, were found chronologically, while the video segment reflects data intervals selected for their relative lack of movement artefacts.

**From/To**	**Effect sizes**					
	**1**	**2**	**3**	**4**	**5**	**Video**
**Fp1 → Fp2**	0.998	1.523	1.462	1.219	1.343	1.382
**Fp1 ← Fp2**	0.775	1.430	1.477	1.578	1.601	2.110
**Fp1 → F4**	0.850	2.281	1.226	1.312	1.355	2.279
**Fp1 ← F4**	0.992	1.863	0.924	1.249	1.091	1.131
**F3 → F4**	0.756	1.294	0.668	0.835	0.821	0.511
**F3 ← F4**	0.587	1.564	0.893	1.039	0.686	1.373
**F3 → Fp2**	0.774	1.688	0.822	1.030	0.781	1.635
**F3 ← Fp2**	0.782	3.349	1.212	1.544	1.370	1.834

8 Classification

To asses the strength of this approach to discriminate between groups, a simple classification was performed using the J48 decision tree algorithm in the Waikato Environment for Knowledge Analysis (WEKA) software [4].

In the Blackpool data video segment a classification accuracy of 86% was achieved using only F3-F4 coherence and the coupling Fp2 $\rightarrow$ F3 as attributes and leave one out cross validation. Table 9 indicates the confusion matrix for this case.

Supplementary Table 9: Confusion matrix for for Blackpool video segment (ASD=13, CG=9). The columns indicate what the rows were classified as, for example, 8 CG participants were correctly classified as CG, while one was incorrectly classified as ASD via this approach.

	**CG**	**ASD**
**CG**	8	1
**ASD**	2	11

The same attributes were used in the other segments to yield confusion matrices for each of the segments investigated (Supplementary Table. 10).

Supplementary Table 10: Confusion matrix for Blackpool sequential segments (ASD = 13, CG = 9). The columns indicate what the rows were classified as.

	**Seg 1**		**Seg 2**		**Seg 3**		**Seg 4**		**Seg 5**	
	**CG**	**ASD**	**CG**	**ASD**	**CG**	**ASD**	**CG**	**ASD**	**CG**	**ASD**
**CG**	9	0	8	1	9	0	8	1	8	1
**ASD**	5	8	2	11	3	10	6	7	6	7

In the Healthy brain network case the data was split into a younger (5-9) and older (9-15) year-old age range groups, for reasons explained in Section 10.2. Classification analysis was subsequently performed on the older age group, with an accuracy of 80% achieved using only coherence between F3-O2, T3-T4 and T3-F3 as attributes. Table 11 indicates the confusion matrix for this case.

Supplementary Table 11: Confusion matrix for for Blackpool video segment (ASD = 31, CG = 33). The columns indicate what the rows were classified as, for example, 29 CG participants were correctly classified as CG, while four were incorrectly classified as ASD.

	**CG**	**ASD**
**CG**	29	4
**ASD**	9	22

9 Additional results

While the main results pertaining to the p-values are given in the main paper, the associated violin plots and group medians are given below to indicate the distributions of the data for each of the sequential time intervals. Additionally, the results of the power analysis are presented.

9.1 Power

While the focus of this investigation was on the connectivity of the frontal region, a preliminary analysis was also performed to assess the differences in power between groups using the wavelet transform, as outlined in the main body. The differences between groups are presented via the p-values generated with the Wilcoxon rank-sum test, analogous to Tab. 3 in the main manuscript.

As demonstrated in Tab. 12, none of the power analyses yielded significant differences, this is in contrast to the consistent variations in connectivity found across the frontal region. The movement

Supplementary Table 12: Reported p-values for power differences between groups across all segments in the frontal electrodes evaluated across the medium frequency band (3.5-12 Hz). The numbered sections 1-5 were found chronologically, while the video segment reflects data intervals selected for their relative lack of movement artefacts. Bold values indicate a p-value < 0.05.

**Probe**	**Power p-values**					
	**1**	**2**	**3**	**4**	**5**	**Video**
**F3**	0.893	0.593	0.593	0.317	0.285	0.640
**F4**	0.841	0.285	0.689	0.125	0.256	0.385
**Fp1**	0.423	0.947	0.548	0.548	0.689	0.463
**Fp2**	0.463	0.504	0.548	0.463	0.640	1.000

artefacts that often bedevil EEG measurements may be responsible for this, as they have a substantially greater affect on amplitude than phase-based measures.

The power measurements also demonstrated far less consistency across repeats, as demonstrated in Fig. 5.

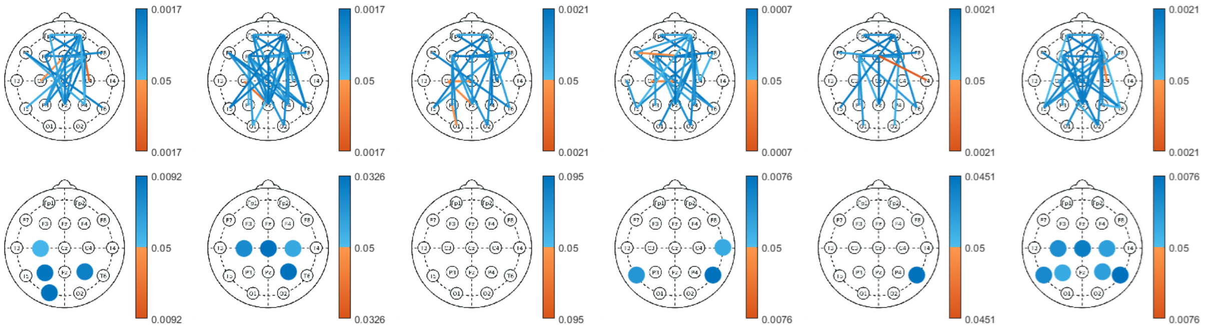

Supplementary Figure 5: The significant ($p < 0.05$, Wilcoxon rank-sum test) coherence (top row) and power (bottom row) differences between groups (ASD = 13, CG = 9) across the sequential and video segments. The columns are in order, so that the first column corresponds to the first segemnt etc, until the sixth column, which represents the video segment.

In each of the repeats, the frontal network demonstrated greater coherence in the video group. In contrast, although the control group generally expressed greater power than the ASD group, the locations of the probes that were significantly different between groups were not consistent across repeats.

9.2 Wavelet phase coherence

Before presenting the violin plots of each segment, the median effective coherence values and associated Friedman test p-value for given probe pairs are indicated. The control group data are given in Tab. 13 and, for comparison, those for the ASD group are given in Tab. 14.

Supplementary Table 13: Median effective coherence values for all segments in the inter-hemispheric frontal network evaluated across the medium frequency band (3.5-12 Hz) for the CG. The numbered sections 1-5 were found chronologically, while the video segments reflect data intervals selected for their relative lack of movement artefacts. The final column gives the Friedman test results.

**From/To**	**Median effective coherence**						**Friedman test**
	**1**	**2**	**3**	**4**	**5**	**Video**	**p-value**
**Fp1 - Fp2**	0.608	0.630	0.628	0.626	0.642	0.635	0.763
**F3 - Fp2**	0.210	0.245	0.224	0.215	0.270	0.205	0.570
**Fp1 - F4**	0.186	0.178	0.202	0.188	0.196	0.181	0.968
**F3 - F4**	0.187	0.204	0.213	0.215	0.204	0.196	0.490

At no point did the p-value from the Friedman test fall below the threshold of 0.05, ensuring that, subject-wise, these measures were consistent across the sequential measurements.

Supplementary Table 14: Median effective coherence values for all segments in the inter-hemispheric frontal network evaluated across the medium frequency band (3.5-12 Hz) for the ASD group. The numbered sections 1-5 were found chronologically, while the video segment reflects data intervals selected for their relative lack of movement artefacts. The final column gives the Friedman test results.

**From/To**	**Median effective coherence**						**Friedman test**
	**1**	**2**	**3**	**4**	**5**	**Video**	***p*-value**
**Fp1 - Fp2**	0.449	0.406	0.399	0.414	0.383	0.403	0.899
**F3 - Fp2**	0.050	0.078	0.073	0.057	0.054	0.086	0.967
**Fp1 - F4**	0.034	0.040	0.044	0.047	0.042	0.037	0.791
**F3 - F4**	0.069	0.058	0.099	0.089	0.053	0.099	0.370

The distributions of the data for each of the sequential segments are given in Figs. 6 - 10.

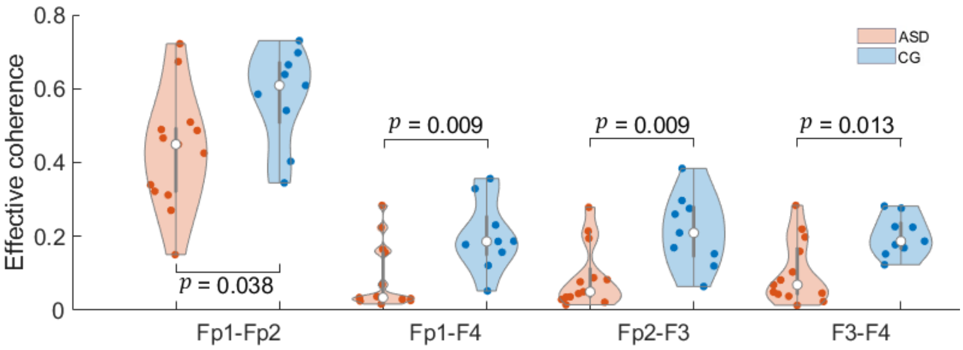

Supplementary Figure 6: Effective coherence values between the measured EEG signal locations for the first sequential segment. The Wilcoxon rank-sum test was used to assess coherence differences between groups ($N = 13$ ASD, $N = 9$ CG). Orange violins represent the ASD group, while blue indicates CG. The white circles illustrate group median values, while the coloured dots represent the effective coherence for each participant in the theta and alpha band between the indicated probe pair.

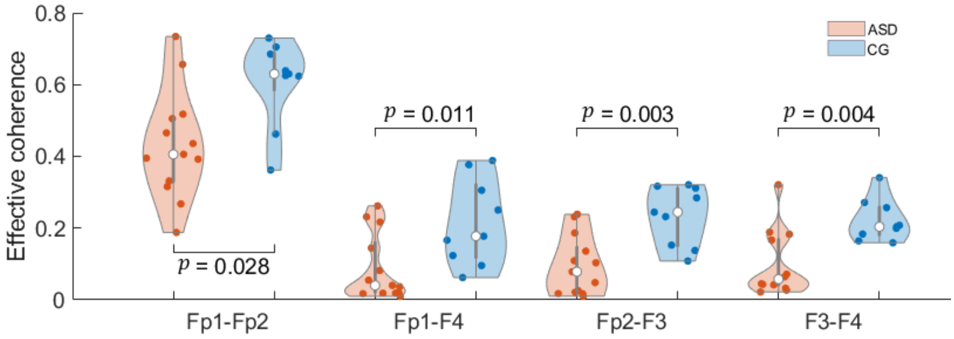

Supplementary Figure 7: Effective coherence values between the measured EEG signal locations for the second sequential segment. Wilcoxon rank-sum test was used to assess coherence differences between groups ($N = 13$ ASD, $N = 9$ CG). Orange violins represent the ASD group, while blue indicates CG. The white circles illustrate group median values, while the coloured dots represent the effective coherence for each participant in the theta and alpha band between the indicated probe pair.

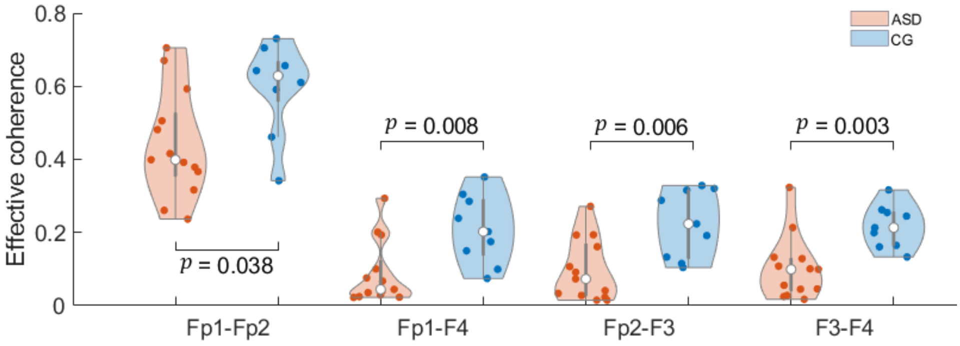

Supplementary Figure 8: Effective coherence values between the measured EEG signal locations for the third sequential segment. Wilcoxon rank-sum test was used to assess coherence differences between groups ($N = 13$ ASD, $N = 9$ CG). Orange violins represent the ASD group, while blue indicates CG. The white circles illustrate group median values, while the coloured dots represent the effective coherence for each participant in the theta and alpha band between the indicated probe pair.

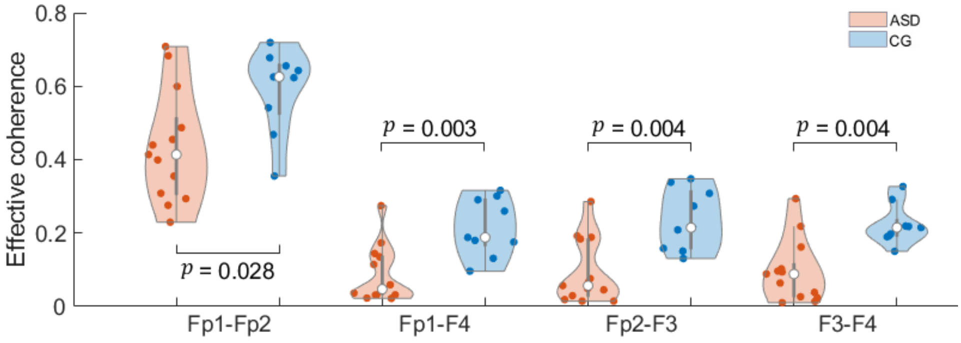

Supplementary Figure 9: Effective coherence values between the measured EEG signal locations for the fourth sequential segment. Wilcoxon rank-sum test was used to assess coherence differences between groups ($N = 13$ ASD, $N = 9$ CG). Orange violins represent the ASD group, while blue indicates CG. The white circles illustrate group median values, while the coloured dots represent the effective coherence for each participant in the theta and alpha band between the indicated probe pair.

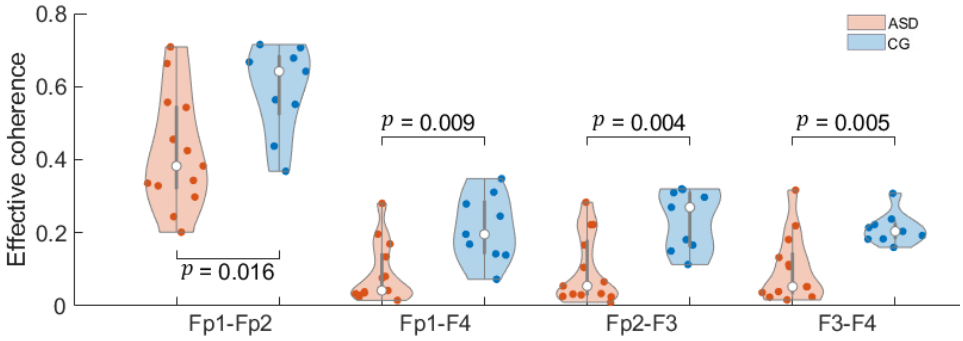

Supplementary Figure 10: Effective coherence values between the measured EEG signal locations for the fifth sequential segment. Wilcoxon rank-sum test was used to assess coherence differences between groups ($N = 13$ ASD, $N = 9$ CG). Orange violins represent the ASD group, while blue indicates CG. The white circles illustrate group median values, while the coloured dots represent the effective coherence for each participant in the theta and alpha band between the indicated probe pair.

9.3 Dynamical Bayesian inference

As with the WPC, we also illustrate the median values of the coupling time between probes. First we consider the control group (Tab. 15) and then the ASD group (Tab. 16).

Supplementary Table 15: Median coupling duration across retests for frontal networks evaluated across the medium frequency band (3.5-12 Hz) for the control group. The numbered sections 1-5 were found chronologically, while the video segment reflects data intervals selected for their relative lack of movement artefacts. The final column indicates the Friedman test p-values, none of which were significant

**From/To**	**Coupling duration (%)**						**Friedman test**
	**1**	**2**	**3**	**4**	**5**	**Video**	p-value
**Fp1 → Fp2**	98.2	98.2	98.2	98.2	96.5	98.2	0.782
**Fp1 ← Fp2**	98.2	96.5	98.2	100	96.5	98.2	0.478
**Fp1 → F4**	22.8	33.3	33.3	33.3	31.6	38.6	0.896
**Fp1 ← F4**	29.8	28.1	22.8	29.8	22.8	22.8	0.800
**F3 → F4**	24.6	24.6	19.3	21.1	24.6	14.0	0.360
**F3 ← F4**	12.3	22.8	24.6	21.1	22.8	26.3	0.999
**F3 → Fp2**	22.8	29.8	14.0	26.3	28.1	15.8	0.793
**F3 ← Fp2**	29.9	43.9	33.3	35.1	35.1	47.4	0.732

At no point did the p-value from the Friedman test fall below the threshold of 0.05, ensuring that, subject-wise, these measures were consistent across the sequential measurements.
The average time coupled between frontal probes in the ASD group was consistently reduced in each sequential segment. As such, the rank-sum test was employed to assess if the difference was statistically significant across the couplings investigated. Figs. 11 - 15 give this p-value for each segment. A table containing all p-values is given in the main text. The violin plots also demonstrate the distributions of the data.

Supplementary Table 16: Median coupling duration across retests for frontal networks evaluated across the medium frequency band (3.5-12 Hz) for the ASD group. The numbered sections 1-5 were found chronologically, while the video segment reflects data intervals selected for their relative lack of movement artefacts. The final column indicates the Friedman test p-values, none of which were significant

**From/To**	**Coupling duration (%)**						**Friedman test**
	**1**	**2**	**3**	**4**	**5**	**Video**	p-value
**Fp1 → Fp2**	57.9	50.9	61.4	70.2	59.6	54.4	0.332
**Fp1 ← Fp2**	71.9	70.2	63.2	66.7	61.4	66.7	0.612
**Fp1 → F4**	8.77	5.26	12.3	8.77	5.26	7.02	0.622
**Fp1 ← F4**	12.3	7.02	8.77	7.02	8.77	10.5	0.811
**F3 → F4**	12.3	8.77	14.0	12.3	10.5	12.3	0.835
**F3 ← F4**	7.02	8.77	12.3	15.8	12.3	8.77	0.834
**F3 → Fp2**	14.0	10.5	8.77	12.3	12.3	10.5	0.795
**F3 ← Fp2**	14.0	8.77	8.77	8.77	8.77	10.5	0.520

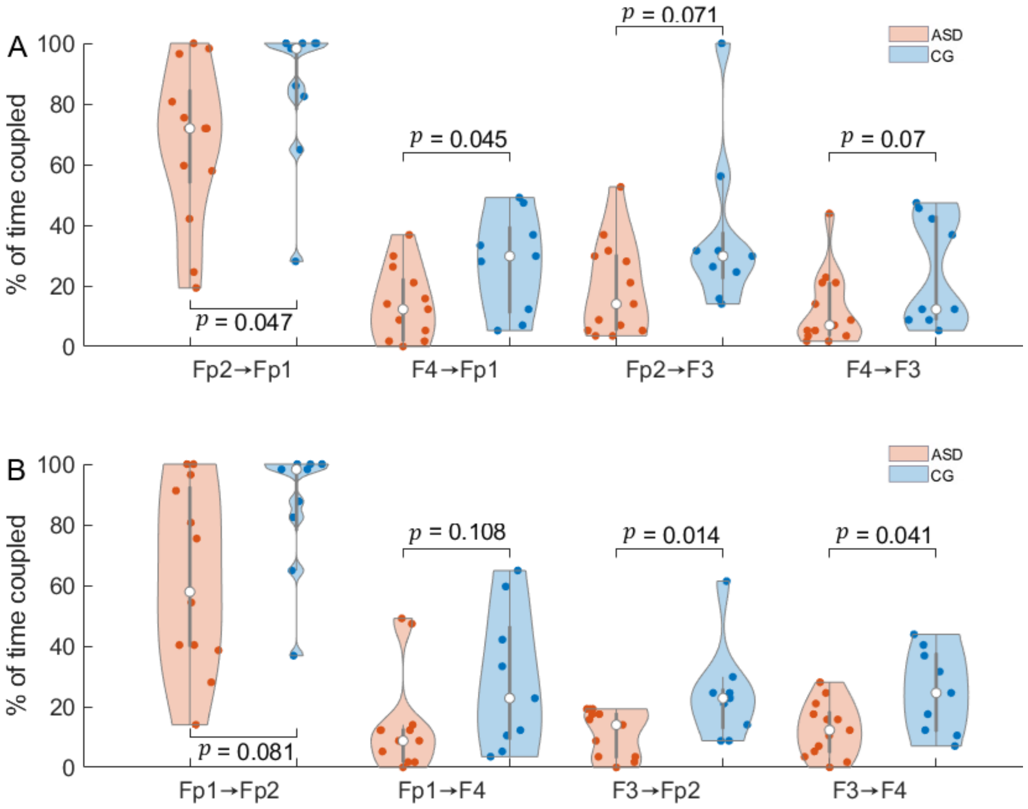

Supplementary Figure 11: Percentage time coupled between EEG probes in the first sequential segment. Wilcoxon rank-sum test was used to assess coupling time differences between groups (N = 13 ASD, N = 9 CG). A) Signals from the left to the right hemisphere. B) Signals from the right to the left hemisphere. p-values indicating differences between the groups are indicated in the figures. Blue violins represent CG while orange indicates ASD. The white circles illustrate group median values, while the coloured dots represent the coupling time for each participant in the theta and alpha band between the indicated probe pair.

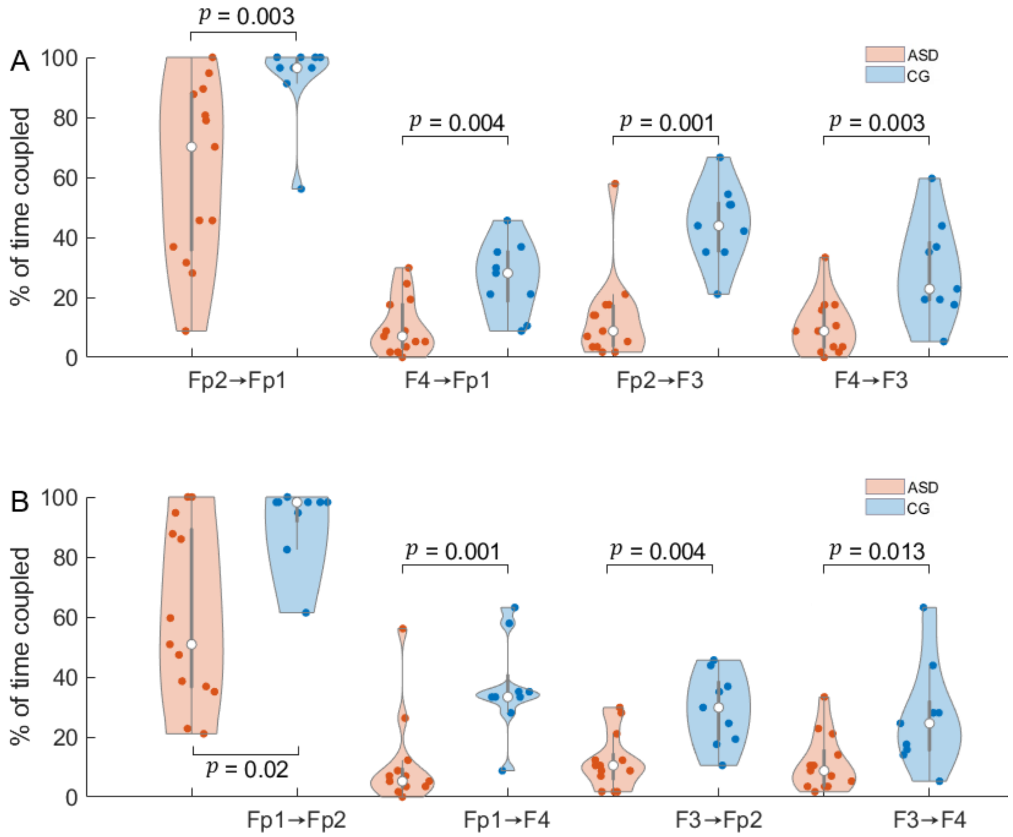

Supplementary Figure 12: Percentage time coupled between EEG probes in the second sequential segment. Wilcoxon rank-sum test was used to assess coupling time differences between groups ($N = 13$ ASD, $N = 9$ CG). A) Signals from the left to the right hemisphere. B) Signals from the right to the left hemisphere. p-values indicating differences between the groups are indicated in the figures. Blue violins represent CG while orange indicates ASD. The white circles illustrate group median values, while the coloured dots represent the coupling time for each participant in the theta and alpha band between the indicated probe pair.

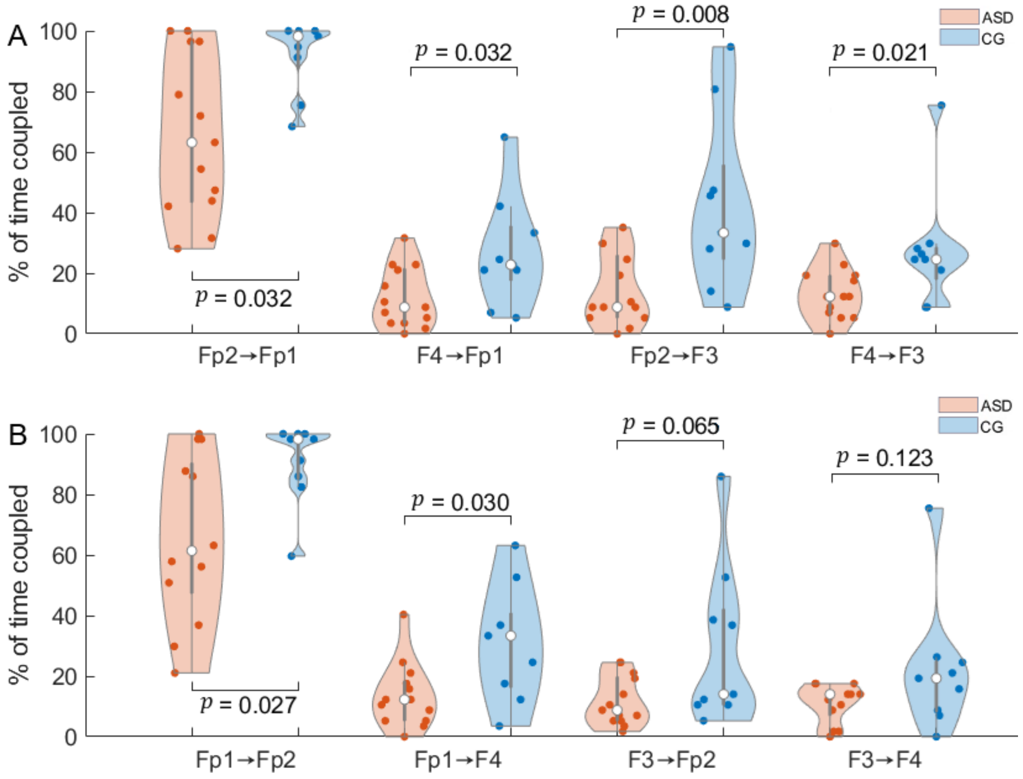

Supplementary Figure 13: Percentage time coupled between EEG probes in the third sequential segment. Wilcoxon rank-sum test was used to assess coupling time differences between groups ($N = 13$ ASD, $N = 9$ CG). A) Signals from the left to the right hemisphere. B) Signals from the right to the left hemisphere. p-values indicating differences between the groups are indicated in the figures. Blue violins represent CG while orange indicates ASD. The white circles illustrate group median values, while the coloured dots represent the coupling time for each participant in the theta and alpha band between the indicated probe pair.

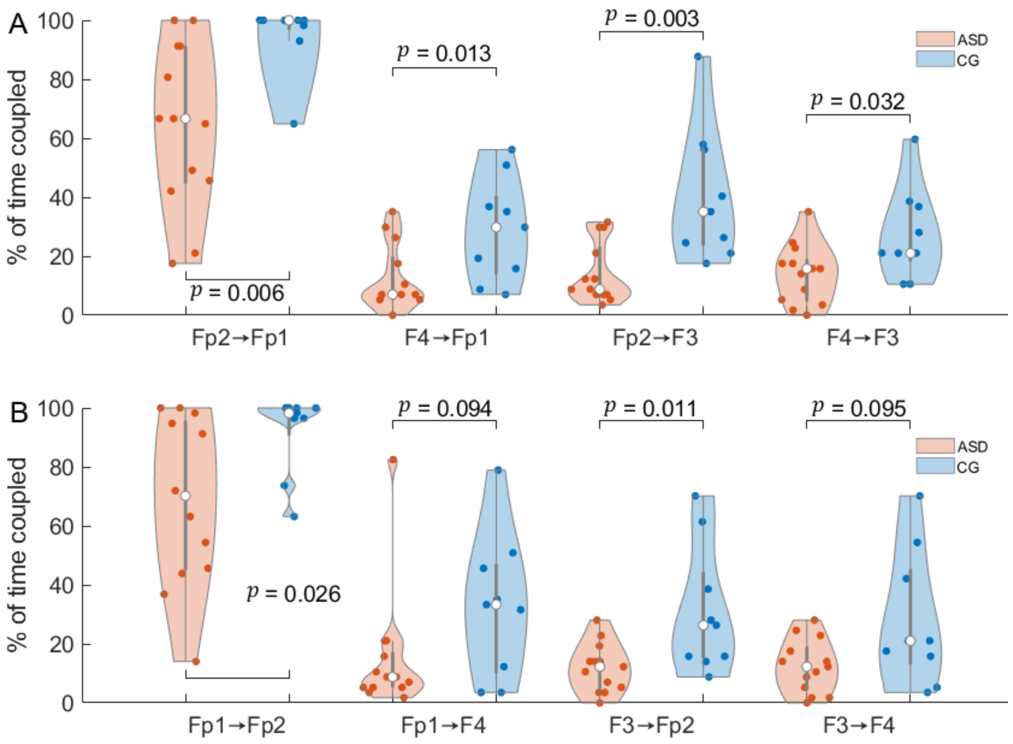

Supplementary Figure 14: Percentage time coupled between EEG probes in the fourth sequential segment. Wilcoxon rank-sum test was used to assess coupling time differences between groups ($N = 13$ ASD, $N = 9$ CG). A) Signals from the left to the right hemisphere. B) Signals from the right to the left hemisphere. p-values indicating differences between the groups are indicated in the figures. Blue violins represent CG while orange indicates ASD. The white circles illustrate group median values, while the coloured dots represent the coupling time for each participant in the theta and alpha band between the indicated probe pair.

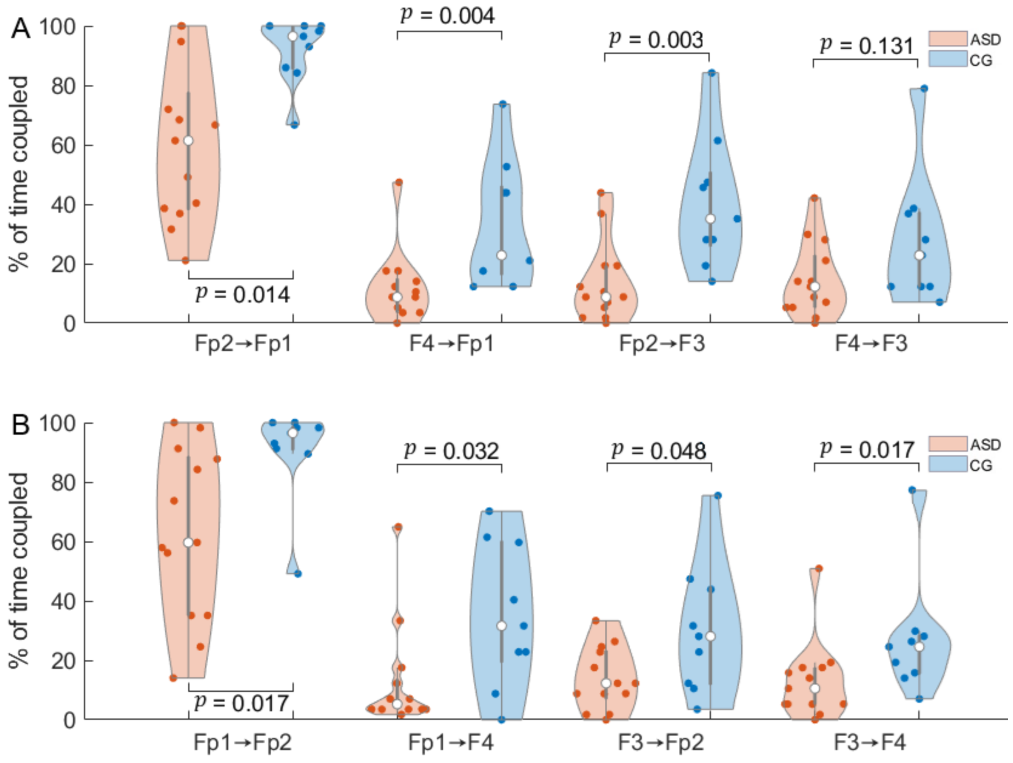

Supplementary Figure 15: Percentage time coupled between EEG probes in the fifth sequential segment. A Wilcoxon rank-sum test was used to assess coupling time differences between groups ($N = 13$ ASD, $N = 9$ CG). A) Signals from the left to the right hemisphere. B) Signals from the right to the left hemisphere. p-values indicating differences between the groups are indicated in the figures. Blue violins represent CG while orange indicate ASD. The white circles illustrate group median values, while the coloured dots represent the coupling time for each participant in the theta and alpha band between the indicated probe pair.

10 Healthy Brain Network

Additional analysis performed on the Healthy Brain Network (HBN) dataset is provided here, including regression and further comparisons across narrow age ranges. Details of the measurements, including inclusion criteria are also outlined. The statistical breakdown of age, IQ and handedness in the ASD and control groups are given in Tab. 2 of the main text.

10.1 Inclusion criteria

As the data were derived from an external source, a number of checks were necessary prior to analysis to ensure they were of sufficient quality. First, the wavelet transforms of each time series recorded, from each probe of interest, were calculated for each individual. This step identified measurement errors that had occurred during data collection. The subsequent corrupted datasets were disregarded.

An example of a clearly nonphysical measurement is illustrated in Fig. 16. The average power was orders of magnitude greater when measurement errors had taken place, and so this was selected as the primary exclusion criterion.

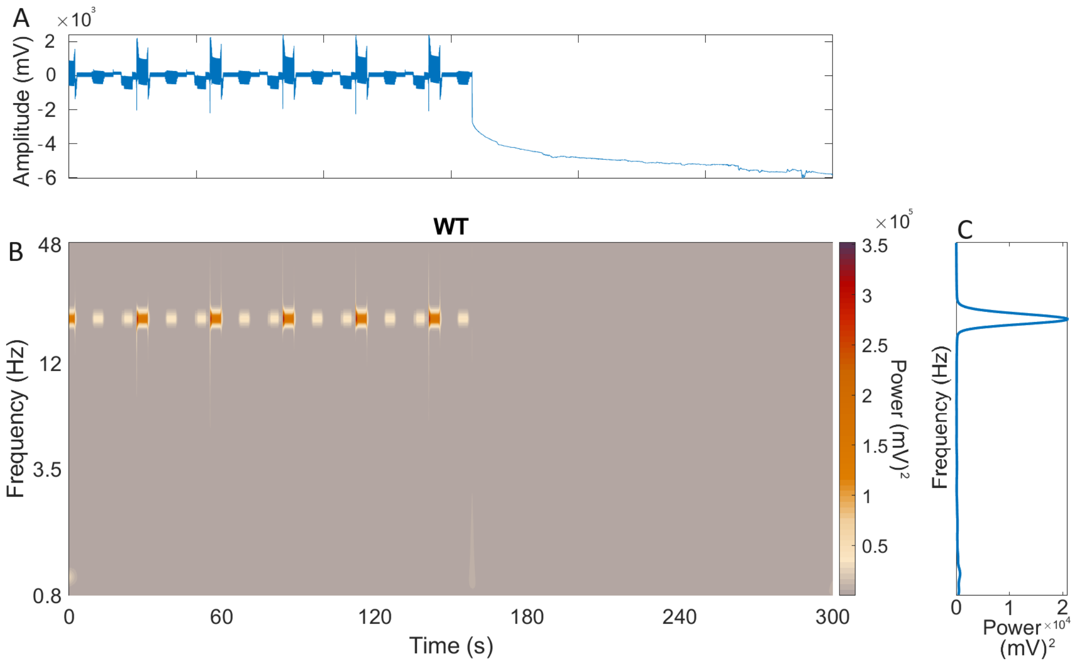

Supplementary Figure 16: EEG data where measurement errors had taken place. A) Time series of the data recorded over 5 minutes. B) Time frequency representation of an EEG signal from the measured data. C) Time-averaged power.

Finally, the groups were selected to ensure IQ, age and handedness were matched so that there were no significant differences ($p < 0.05$) between groups following the application of a Wilcoxon rank-sum test. A statistical breakdown of the phenotypic data within the HBN groups is given in Tab. 2 of the main text.

10.2 Splitting the age groups

The size of the HBN dataset enabled further analysis between two cohorts; one younger (5-9 years) and one older (9-15 years). Here, we compare both the global and local coherence across various age ranges. For comparison, additional analysis of the 5-15 group is also presented.

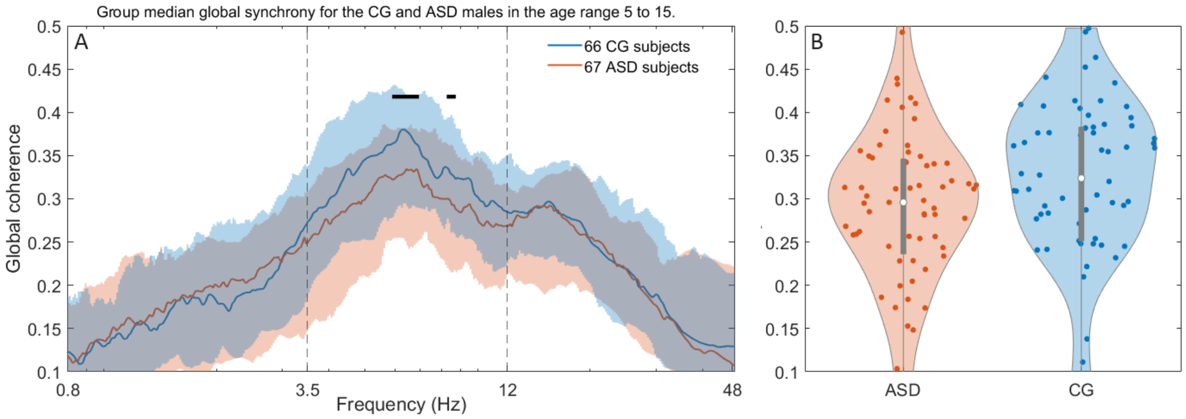

Supplementary Figure 17: Total global coherence for the 5-15 age range. (A) Median total global coherence for the ASD (orange) and CG (blue) individuals. Shaded areas represent the 25th and 75th percentiles, while the solid lines indicate group medians.The Wilcoxon rank-sum test was applied between the groups at each frequency ($N = 67$ ASD, $N = 66$ CG). Black lines plotted above the medians indicate a p - value less than 0.05. In this case, there were no significant differences at any frequency. (B) Violin plot of the mean global coherence across the medium frequency (3.5-12 Hz) region. The median is given by the white circle while the grey box illustrates the interquartile range. Wilcoxon rank-sum test found no significant difference between groups, with $p = 0.0641$

Despite the CG group coherene being higher on average in the MF region, and significantly so at certain frequencies as depicted in Fig. 17A, a rank-sum test across the entire MF band failed to exceed the significance threshold ($p = 0.0641$). The global coherence was also not significantly different ($p = 0.601$) for the 5-9-year-old group (Fig. 18).

There is, however, a significantly greater global coherence in the medium frequency region for the 9-15-year-old age group ($p = 0.029$, Fig. 19).

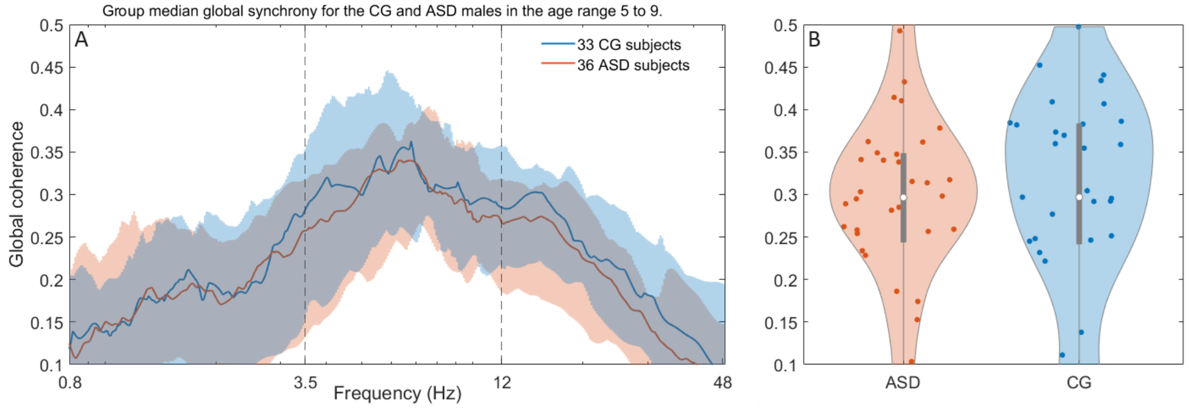

Supplementary Figure 18: Total global coherence for the 5-9 age range. (A) Median total global coherence for the ASD (orange) and CG (blue) individuals. the shaded areas represent the 25th and 75th percentiles while the lines indicate group medians. groups were assessed at each frequency bin using the Wilcoxon rank-sum test ($N = 37$ ASD, $N = 33$ CG). Black lines plotted above the medians indicate a p- value less than 0.05. (B) Violin plot of the average value across the medium frequency (3.5-12Hz) region. the median is given by the withe circle and the box illustrates the interquartile range. A Wilcoxon rank-sum test found no significant difference between groups, with $p = 0.601$

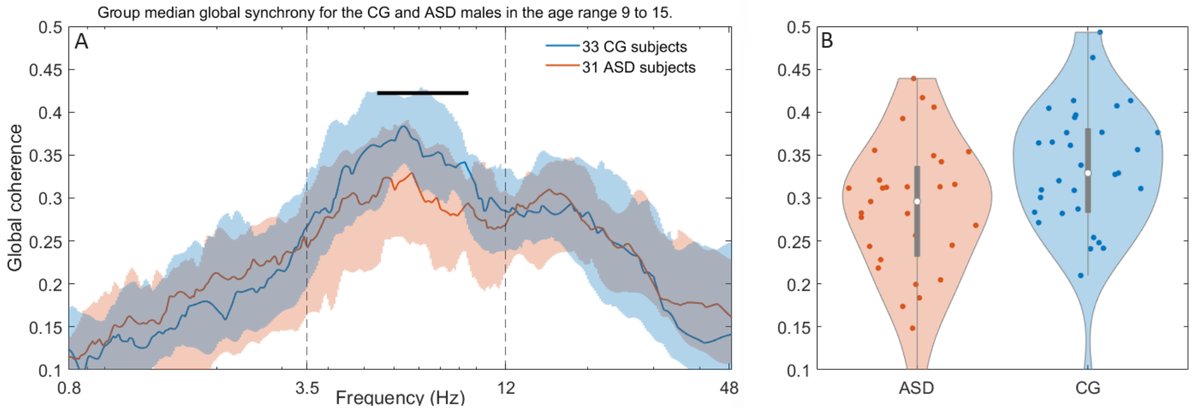

Supplementary Figure 19: Total global coherence for the 9-15 age range. (A) Median total global coherence for the ASD (orange) and CG (blue) individuals. the shaded areas represent the 25th and 75th percentiles while the lines indicate group medians. groups were assessed at each frequency bin using the Wilcoxon rank-sum test ($N = 31$ ASD, $N = 33$ CG). Black lines plotted above the medians indicate a p- value less than 0.05. (B) Violin plot of the average value across the medium frequency (3.5-12Hz) region. the median is given by the withe circle and the box illustrates the interquartile range. AWilcoxon rank-sum test found a significant difference between the groups, with $p = 0.029$

Considering the above results there seems to be a dependence upon age regarding the level of global coherence. As this data was collected externally, it is possible that the younger children were less able to tolerate the battery of tests. As participants must remain still and follow experimental instructions, it is possible that the older group were better able to tolerate the procedure. This may be responsible for the differences in the groups. The 9-15 group comparison supports the conclusion of decreased global connectivity across the entire brain for the medium frequency region.

In the 5-9 group there are also very few local connectivity differences (Fig. 20A). In contrast, both the 5-15 (Fig. 20B) and 9-15 (Fig. 20C) age ranges have many significant differences, with more in the latter (18 and 25 percent of possible connections, respectively). This may be further evidence that the data in the 5-9 age group are obfuscated by a reduced ability to tolerate the measurement procedure.

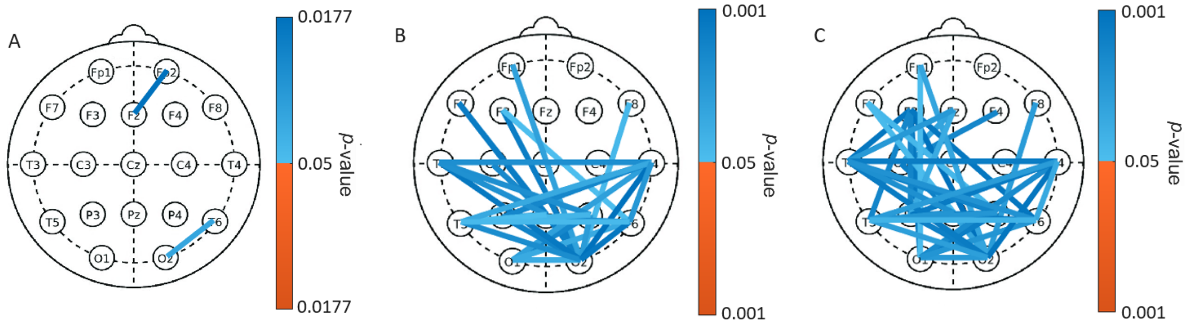

Supplementary Figure 20: Local coherence differences between ASD and CG groups in various age ranges. A Wilcoxon rank-sum test was performed to compare coherence between groups p-values are plotted when the threshold of 0.05 is exceeded. Orange indicates ASD $>$ CG while blue indicates CG $>$ ASD. (A) The 5-9 age range (N = 37 ASD, N = 33 CG), only 2 connections were significantly different, meaning 1.3 % of possible probe pairs. (B) The 5-15 age range (N = 67 ASD, N = 66 CG), 28 connections were significant, 18 percent of the possible combinations. (C) The 9-15 age range (N = 31 ASD, N = 33 CG), 38 connections were significant, 25 percent of the possible combinations.

References

[1] SJK Barnes, J Bjerkan, PT Clemson, J Newman, and A Stefanovska. Phase coherence — A time-localized approach to studying interactions. *Chaos: An Interdisciplinary Journal of Nonlinear Science*, 34(7), 2024.

[2] Jacob Cohen. *Statistical Power Analysis for the Behavioral Sciences.* Academic Press, 2013.

[3] Franz Faul, Edgar Erdfelder, Albert-Georg Lang, and Axel Buchner. G* power 3: A flexible statistical power analysis program for the social, behavioral, and biomedical sciences. *Behavior Research Methods*, 39(2):175–191, 2007.

[4] Eibe Frank, Mark A Hall, and Ian H Witten. *The WEKA workbench.* Morgan Kaufmann, 2016.

[5] Jean-Philippe Lachaux, Eugenio Rodriguez, Michel Le Van Quyen, Antoine Lutz, Jacques Martinerie, and Francisco J Varela. Studying single-trials of phase synchronous activity in the brain. *International Journal of Bifurcation and Chaos*, 10(10):2429–2439, 2000.

[6] Jean-Philippe Lachaux, Antoine Lutz, David Rudrauf, Diego Cosmelli, Michel Le Van Quyen, Jacques Martinerie, and Francisco Varela. Estimating the time-course of coherence between single-trial brain signals: an introduction to wavelet coherence. *Neurophysiologie Clinique/Clinical Neurophysiology*, 32(3):157–174, 2002.

[7] Christopher Torrence and Peter J Webster. Interdecadal changes in the enso–monsoon system. *Journal of climate*, 12(8):2679–2690, 1999.
